# Klyflaccicembranols A–I, New Cembranoids from the Soft Coral *Klyxum flaccidum*

**DOI:** 10.3390/md15010023

**Published:** 2017-01-21

**Authors:** Atallah F. Ahmed, Chia-Ruei Tsai, Chiung-Yao Huang, Sheng-Yang Wang, Jyh-Horng Sheu

**Affiliations:** 1 Department of Marine Biotechnology and Resources, National Sun Yat-sen University, Kaohsiung 80424, Taiwan; afahmed@ksu.edu.sa (A.F.A.); qse456awd@gmail.com (C.-R.T.); huangcy@mail.nsysu.edu.tw (C.-Y.H.); 2 Department of Pharmacognosy, College of Pharmacy, King Saud University, Riyadh 11451, Saudi Arabia; 3 Department of Pharmacognosy, Faculty of Pharmacy, Mansoura University, Mansoura 35516, Egypt; 4 Department of Forestry, National Chung Hsing University, Taichung 40227, Taiwan; taiwanfir@dragon.nchu.edu.tw; 5 Institute of Natural Products, Kaohsiung Medical University, Kaohsiung 80756, Taiwan; 6 Department of Medical Research, China Medical University Hospital, China Medical University, Taichung 40402, Taiwan; 7 Frontier Center for Ocean Science and Technology, National Sun Yat-sen University, Kaohsiung 80424, Taiwan; 8 Doctoral Degree Program in Marine Biotechnology, National Sun Yat-sen University, Kaohsiung 80424, Taiwan

**Keywords:** soft coral, *Klyxum flaccidum*, cembranoid, NO inhibition, cytotoxicity

## Abstract

New cembranoids klyflaccicembranols A–I (**1**–**9**), along with gibberosene D (**10**), have been isolated from the organic extract of a Formosan soft coral *Klyxum flaccidum*. Their structures were established by extensive spectroscopic analyses, including 2D NMR spectroscopy, and spectral data comparison with related structures. The cytotoxicity of the isolated metabolites, as well as their nitric oxide (NO) inhibitory activity, were evaluated and reported. Metabolites **2**, **4**, **6**, **8** and **9** were found to exhibit variable activities against a limited panel of cancer cell lines in a range of IC_50_ 16.5–49.4 μM. Among the tested cembranoids, compounds **4**, **5**, **6**, and **9** significantly inhibited NO production in lipopolysaccharide (LPS)-stimulated RAW264.7 macrophages at a dose of 50 μg/mL.

## 1. Introduction

The marine environment has long been recognized as an exceptional source of new natural products with a diverse range of chemical structures and bioactivities, including anti-cancer, antiviral, immunosuppressive and anti-inflammatory activities [1,2,3]. This structural diversity has supplied unique chemicals for the pharmaceutical, cosmetics, and even agrochemicals industry [4]. Soft corals belonging to the genus *Klyxum* (Alcyoniidae), including *Klyxum flaccidum*, are considered rich sources of eunicellin-based diterpenoids species [2,5,6,7,8,9,10], of which many compounds have been found to exert anti-inflammatory [7,8,9,10] and cytotoxic [8,11] effects. A series of cytotoxic and anti-inflammatory steroids was also discovered from *K. flaccidum* in our previous investigations [12,13]. Cembranoid-based diterpenoids have not hitherto been isolated from soft corals of the genus *Klyxum*. However, our continuing investigation into the chemical constituents of a Formosan soft coral *K. flaccidum* has led to the discovery of a series of new polyoxygenated cembranoids. Extensive spectroscopic analyses, including 2D NMR spectroscopy and spectral comparison, were applied to establish the structures of these new metabolites. The cytotoxicity of the isolated metabolites was further assessed against a limited panel of cancer cell lines via Alamar Blue assay [14,15], and anti-inflammatory activity was evaluated in terms of their potential to inhibit nitric oxide (NO) production in lipopolysaccharide (LPS)-stimulated RAW264.7 macrophages.

## 2. Results and Discussion

**Figure 1:**
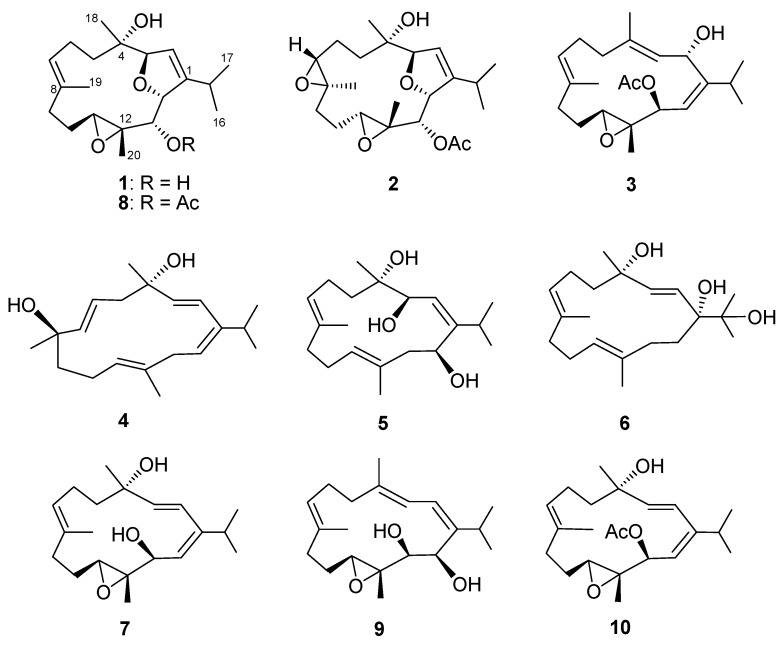
Structures of cembranoids isolated from *Klyxum flaccidum*.

Sliced bodies of the soft coral *K. flaccidum* were extracted exhaustively with ethyl acetate (EtOAc). The solvent-free extract was then fractionated over a silica gel 60 column to yield 26 fractions. Fractions that displayed terpenoidal methyl, olfeinic and oxymethine proton signals in the ^1^H NMR spectrum were selected and further purified using a successive series of silica gel 60 and RP-18 gel columns to yield compounds **1**–**10** (Figure 1). 

**Figure 2:**
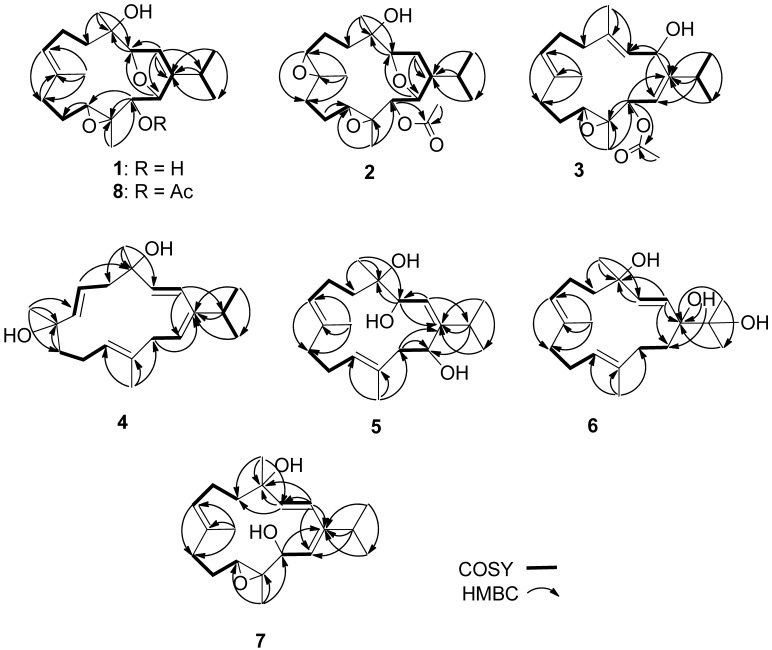
Correlation spectroscopy (COSY) and heteronuclear multiple bond correlation (HMBC) correlations in **1**–**8**.

Klyflaccicembranol A (**1**) was isolated as a colorless oil. Its molecular formula C_20_H_32_O_4_ was deduced from the sodium adduct ion peak (*m*/*z* 359.2196 [M + Na]^+^) in the high-resolution electrospray ionization mass spectrometry (HRESIMS) and NMR data measured in C_6_D_6_ (Table 1 and Table 2). Thus, five degrees of unsaturation were indicated. The infrared (IR) absorption band at ν_max_ 3445 cm^−1^ revealed the presence of hydroxy functionality. The ^13^C NMR spectrum displayed 20 carbon signals (Table 1), which were characterized by distortionless enhancement by polarization transfer (DEPT) and heteronuclear single-quantum correlation (HSQC) spectra as five methyls, four methylenes, seven methines (including two olfeinic and four *sp*^3^ oxymethines), and four nonprotonated carbons (including two olfeinic and two *sp*^3^ oxycarbons). The NMR signals at δ_C_ 147.8, C; 132.1, C; 127.0, CH and 121.0, CH; δ_H_ 5.59, 1H, br s and 5.38, 1H, dd, *J* = 5.2, 5.2 Hz indicated the presence of two trisubstituted double bonds. Thus, the remaining three unsaturations suggested **1** to be a tricyclic diterpenoid. However, it was found that the NMR data of **1** differed from those of eunicellins discovered from *Klyxum* species [2,5,6,7,8,9,10] and resembled those of cembranoids isolated from *Sinularia* and *Sarcophyton* species [16,17,18], as no correlation spectroscopy (COSY) correlated ring-juncture *sp*^3^ methine protons were detected in **1**. One trisubstituted epoxide (δ_C_ 61.9, C and 59.7, CH; δ_H_ 3.15, 1H, dd, *J* = 6.4, 2.0 Hz) and an ether bridge (δ_C_ 89.7, CH; δ_H_ 4.61, 1H, d, *J* = 4.8 Hz and δ_C_ 85.8, CH; δ_H_ 4.78, 1H, br d, *J* = 4.8 Hz) of a 2,3,5-trisubstituted dihydrofuran moiety [18] were also deduced. Thus, the remaining two oxygen atoms of the molecular formula were ascribed to two hydroxy groups linked to the carbon atoms resonating at 72.8 (CH) and 74.2 (C). Analysis of the COSY correlations of **1** indicated the presence of four proton-correlated partial structures, including those of an isopropyl group (Figure 2). By analysis of heteronuclear multiple bond correlation (HMBC) correlations observed from the methyl, olfeinic, and oxymethine protons, it was possible to connect the four partial structures and to assign the positions of the two hydroxy groups, one trisubstituted double bond, and a trisubstituted epoxide at C-4, C-13, C-7/C-8, and C-11/C-12, respectively (Figure 2). The ether linkage of the dihydrofuran was confirmed by the HMBC correlations found from H-2 (δ_H_ 5.59, br s) to C-1 (δ_C_ 147.8, C), C-3 (δ_C_ 89.7, CH) and C-14 (δ_C_ 85.8, CH) and from H-14 (δ_H_ 4.78, br d, *J* = 4.8 Hz) to C-3, respectively. The planar structure of compound **1** was thus established as 4,13-dihydroxy-3,14:11,12-bisepoxy-cembra-1,7-diene. 

**Figure 3:**
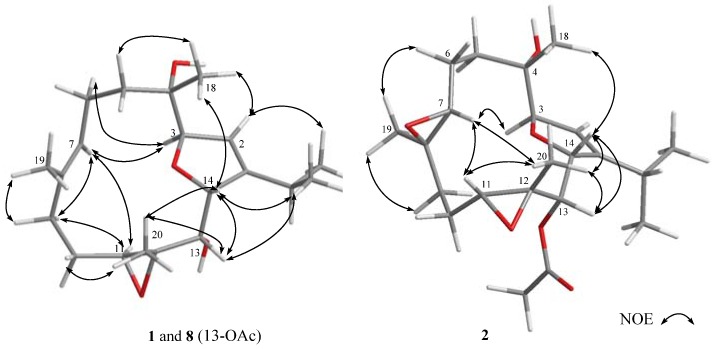
Key nuclear Overhauser effect ‎(NOE) correlations of **1**, **8** and **2**.

The relative configuration of **1** was determined by the analysis of nuclear Overhauser effect (NOE) correlations in a nuclear Overhauser enhancement spectroscopy (NOESY) experiment and with the assistance of ^5^*J*_H,H_ coupling constants, in addition to molecular modeling using molecular mechanical parameters (MM2 force field) calculations (Figure 3). The high magnitude of the long-range coupling constant of H-3 and H-14 (*J* = 4.8 Hz) of the 2,5-dihydrofuran ring in **1** suggested the *trans* orientation of protons at C-3/C-14 [19]. Accordingly, no NOE correlation was detected between H-3 and H-14. From the NOESY spectrum of **1**, it was found that H_3_-18 showed NOE interaction with H-14, and H-14 with H-13 and H_3_-20; therefore, due to the *β-*orientation of H_3_-18, H-13 and H-14 should also be positioned on the *β*-face. Furthermore, H-3 exhibited NOE correlation with the olefinic H-7, as did H-7 with H-11, while H-11 did not show NOE interaction with H_3_-20, revealing that H-3 and H-11 should be positioned on the *α*-face. Moreover, the NOE interactions exhibited for the olefinic H-2 with H_3_-17 and for H_3_-19 with H-9, but not with H-7, enabled the assignment of *Z* and *E* geometries of the double bonds at C-1/C-2 and C-7/C-8, respectively. As a result, the (3R*,4S*,11S*,12R*,13S*,14R*)-configuration of **1** was established.

**Figure 4:**
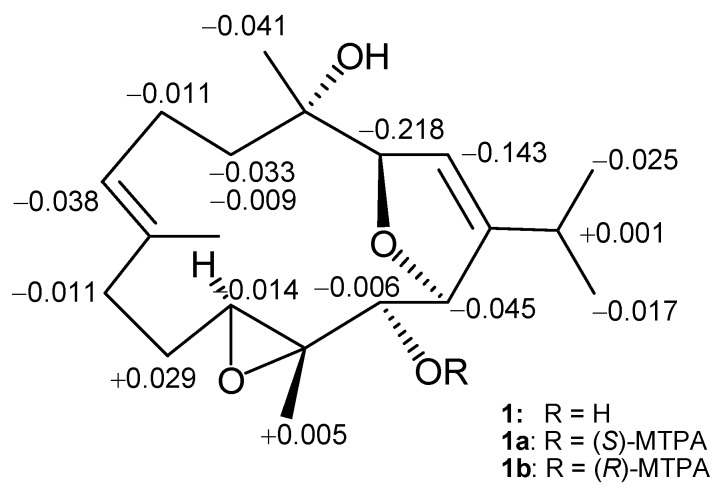
^1^H NMR chemical shift differences ∆δ (δ*S* − δ*R*) in ppm for the α-methoxy-α-(trifluoromethyl)phenylacetic ‎(MTPA) esters of **1**.

After isolation and chemical identification of **1**, related compound klyflaccicembranol H (**8**) was isolated from the less polar fraction of the same extract of the organism. Compound **8** possessed the molecular formula C_22_H_34_O_5_, as indicated by the HRESIMS (*m*/*z* 378.2404 [M]^+^) and NMR data (Table 1 and Table 3). IR absorptions at ν_max_ 3310 and 1730 cm^−1^ and electron ionization mass spectroscopy EIMS ion peaks at *m*/*z* 318 [M − AcOH]^+^ and *m*/*z* 300 [M − AcOH − H_2_O]^+^ revealed the presence of hydroxy and acetoxy functionalities in the molecule of **8**. On comparison of spectroscopic data, the NMR spectrum of **8** was found to be very similar to that of **1** (Table 1, Table 2 and Table 3), with the exception of the presence of signals of an additional methyl and an ester carbonyl carbon of an acetoxy group at δ_C_ 169.9, C and 20.6, CH_3_; and δ_H_ 2.06, 3H, s. These were associated with a downfield shift displayed by H-13 in **8** (δ_H_ 5.16, d, *J* = 2.0 Hz) relative to that of **1** (δ_H_ 3.58, br s). Therefore, **8** was suggested to be the 13-acetylated derivative of **1**, as proven by complete 2D NMR correlations analyses (Figure 2). Moreover, compound **8**, measured in CDCl_3_, showed the same NOE correlations that had been observed in the NOESY spectrum of **1**, indicating the same relative configuration. Furthermore, hydrolysis of **8** yielded **1**. To clarify the absolute configuration at C-13 in **1** (and hence also in **8**), a modified Mosher’s method [20,21] was employed to prepare (*S*)- and (*R*)-α-methoxy-α-(trifluoromethyl)phenylacetic (MTPA) esters of **1** (**1a** and **1b**, respectively) using the corresponding (−)- and (+)-MTPA-chloride, respectively. Calculation of ∆δ values (δ_H_ *S* − δ_H_ *R*) for the protons adjacent to C-13 led to the assignment of the *S* configuration at C-13 in **1**, and consequently therefore in **8** (Figure 4). On the basis of the above findings and detailed NOESY correlations analyses (Figure 3), compound **1** was identified as (3*R*,4*S*,11*S*,12*R*,13*S*,14*R*,1*Z*,7*E*)-4,13-dihydroxy-3,14:11,12-bisepoxy-cembra-1,7-diene. Compound **8** (klyflaccicembranol H) was thus subsequently characterized as the C-13 acetyl derivative of **1**.

Klyflaccicembranol B (**2**) was also isolated as a colorless oil. The sodium adduct ion peak [M + Na]^+^ appearing at *m*/*z* 417.2250 in the HRESIMS was appropriate for the molecular formula C_22_H_34_O_6_ with six unsaturations. The IR absorption bands at ν_max_ 3443 and 1746 cm^−1^ indicated hydroxy and ester functionalities in the molecule. The NMR signals (δ_C_ 169.9, C and 21.0, CH_3_; δ_H_ 1.91, 3H, s) showed the ester functionality to be an acetoxy group. A trisubstituted-2,5-dihydrofuran moiety (δ_C_ 147.7, C; 121.2, CH; 91.1, CH and 85.0, CH; δ_H_ 5.65, br s; 5.05, d, *J* = 5.0 Hz and 4.78, d, *J* = 5.0 Hz) was also deduced, as in case of **1** and **8**. However, the ^13^C NMR spectral comparison of compound **2** with **8** in the regions of δ_C_ 125–140 and 58–65 ppm indicated that **2** had an additional trisubstituted epoxide, instead of the 7,8-trisubstiututed double bond in **8**. The gross structure of **2** was thus established by analysis of COSY and HMBC correlations (Figure 2). The relative configuration of **2** was determined by analysis of NOE correlations, as shown in Figure 3. The NOE interactions observed for H_3_-19 (δ_H_ 1.24, s) with H-6 (δ_H_ 1.59, m), H-7 (δ_H_ 3.07, dd,* J* = 6.0, 2.5 Hz) with H-3, and H-11 (δ_H_ 2.98, d, *J* = 7.5 Hz) with H_3_-20 (δ_H_ 1.42, s), indicated the *trans* and *cis* geometries of the trisubstituted 7,8- and 11,12-epoxides, respectively. As H-3 exhibited a NOE correlation with H-7, H-7 and H_3_-19 were thus α- and β-oriented, respectively. Moreover, the NOE correlations observed for H-14 with both H_3_-20 and H-13 disclosed the α-orientation of the acetyl group at C-13 and the β-orientation of the methyl group at C-12, and hence the β-oriented epoxide proton at C-11. On the basis of the above findings and the absolute configuration of biogenetically-related **1**, compound **2** was thus determined as (3*R*,4*S*,7*R*,8*R*,11*R*,12*R*,13*S*,14*R*,1*Z*)-7,8:11,12:3,14-triepoxy-4-hydroxy-13- acetoxycembra-1-ene.

**Figure 5:**
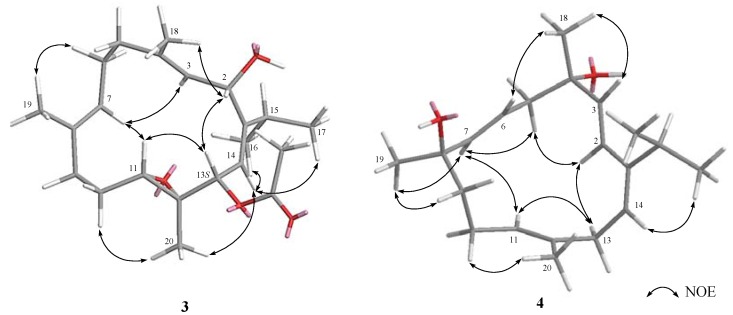
Key NOE correlations of **3** and **4**.

New metabolite **3** was found to have the molecular formula C_22_H_34_O_4_, as deduced from the HRESIMS (*m*/*z* 385.2354 [M + Na]^+^) and NMR data (Table 1 and Table 2), implying six degrees of unsaturation. As in the cases of **2** and **8**, the IR absorptions at 1731 and 3450 cm^−1^ further indicated the presence of an ester moiety and a hydroxy group. The NMR data (Table 1 and Table 2) of an acetoxy group (δ_C_ 170.7, C; 21.1, CH_3_; δ_H_ 2.10, 3H, s), a trisubstituted epoxide (δ_C_ 61.3, C and 59.7, CH) and three trisubstituted olefins (δ_C_ 155.9, 137.6, 133.2, each C, and 126.3, 125.2, 115.6, each CH) established five degrees of unsaturation in the molecule. By comparison of NMR spectroscopic data with those of gibberosene D (**10**) isolated from the soft coral *S. gibberosa* [16], compound **3** was suggested to be a regioisomer of **10**. Interpretation of 2D NMR correlations (Figure 2) indicated a secondary hydroxy group at C-2 and a trisubstituted double bond at C-3/C-4 in **3** instead of the disubstituted double bond and the tertiary hydroxy group at C-2/C-3 and C-4, respectively, in **10**. Detailed COSY and HMBC correlations (Figure 2) further determined the C-7/C-8, C-1/C-14, C-11/C-12, and C-13 positions for the two other olefins, the epoxide, and the acetoxy group in the structure of **3**, respectively, as were identified in **10** (Figure 1). Thus, the gross structure of **3** was established as 13-acetoxy-11,12-epoxy-2-hydroxycembra-3,7,14-triene. The NOE interaction of H-13 with H-2 enabled assignment of their *syn* positions, and hence the α-orientation of the hydroxy group at C-2, based on the previously defined *S* configuration at C-13 (as in **1** and **8**). The NOE interactions of H-14 with H_3_-20 (δ_H_ 1.22, 3H, s) and H-13 with H-11 (δ_H_ 2.47, dd, *J* = 7.2, 2.0 Hz) indicated the *trans* geometry of the 11,12-epoxide, and, consequently, the 11*S* and 12*R* configurations. Furthermore, other NOE interactions of H-2 with H_3_-18 (δ_H_ 1.80, 3H, s), H-3 with H-7 (δ_H_ 4.83, br d, *J* = 6.0 Hz), and H_3_-19 (δ_H_ 1.55, 3H, s) with H-6 (δ_H_ 2.27, m) established the *E* geometries of the C-3/C-4 and C-7/C-8 double bonds. The *Z* geometry of the double bond at C-14/C-1 in **3** was proven by the NOE correlation (Figure 5) of H-14 with the isopropyl methyls at C-15 (δ_H_ 1.05 and 1.09, each 3H, d, *J* = 6.8 Hz). The above observations thus established the structure of klyflaccicembranol C (**3**) as (2*S*,11*S*,12*R*,13*S*,3*E*,7*E*,14*Z*)-13-acetoxy-11,12-epoxy-2-hydroxycembra-3,7,14-triene.

New metabolite klyflaccicembranol D (**4**) was isolated as a pale oil. A pseudomolecular ion peak [M + Na]^+^ at *m*/*z* 327.2200 in the HRESIMS data was observed, corresponding to the molecular formula C_20_H_32_O_2_ and five degrees of unsaturation. The IR absorption at ν_max_ 3419 cm^−1^ and two *sp*^3^ oxycarbon signals appearing at δ_C_ 72.9 and 72.5 indicated the hydroxyl-bearing characteristic of the compound. Two trisubstituted double bonds were also identified from the NMR signals at δ_C_ 146.3, C; 132.8, C; 126.7, CH; 122.0, CH; δ_H_ 5.48 (1H, dd, *J* = 8.0, 5.6 Hz), and 5.11 (1H, dd, *J* = 7.2, 7.2 Hz). Analysis of COSY correlations of **4** established five consecutive proton sequences, including those of the isopropyl group (Figure 2). Careful study of the HMBC spectrum of **4** further established the connectivities of the five proton sequences with the diagnostic nonprotonated carbons (C-1, C-4, C-8, and C-12), as illustrated in Figure 2. Thus, the two 1,2-disubstuituted and the two trisubstituted double bonds were localized at C-2/C-3, C-6/C-7, C-11/C-12, and C-14/C-1, respectively, whereas the hydroxy groups were positioned at C-4 and C-8. Thus, the planar structure of **4** was established as 4,8-dihydroxycembra-2,6,11,14-tetraene. The large coupling constants of *J*_2,3_ (16.4 Hz) and *J*_6,7_ (15.6 Hz) reflected the *E* geometries of the two disubstituted double bonds at C-2/C-3 and C-6/C-7. Moreover, the NOE correlations (Figure 5) found for H_3_-20 (1.60, 3H, s) with one proton of H_2_-10 (δ_H_ 2.37, m), but not with H-11, and for H-14 with the protons of the two methyls at C-15, indicated the *E* and* Z* geometries of the double bonds at C-11/C-12 and C-1/C-14, respectively. On the basis of the previously defined α-orientation of 4-OH in biogenetically-related compounds **1**, **2**, and **8**, and the NOE interactions of the β-oriented H_3_-18 (δ_H_ 1.27, 3H, s) with the olefinic H-6 and the *trans* downward-oriented H-7 with H_3_-19 (δ_H_ 1.16, 3H, s), the β-orientation of the hydroxy group at C-8 was proven. These findings, together with detailed analysis of other NOE correlations (Figure 4), indicated the structure of klyflaccicembranol D (**4**) to be (4S,8*R*,2*E*,6*E*,11*E*,14*Z*)-4,8-dihydroxycembra-2,6,11,14-tetraene.

**Figure 6:**
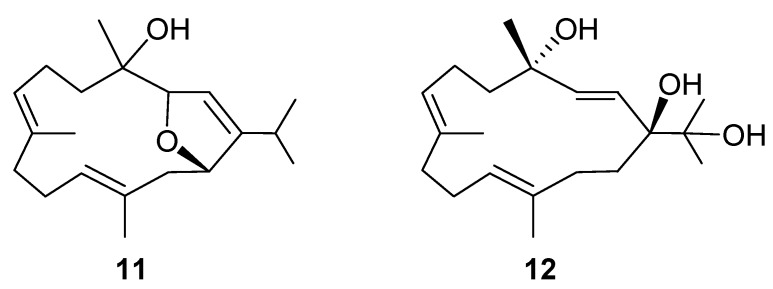
Structures of semisynthetic cembranoid (**11**) and crassumol A (**12**).

**Figure 7:**
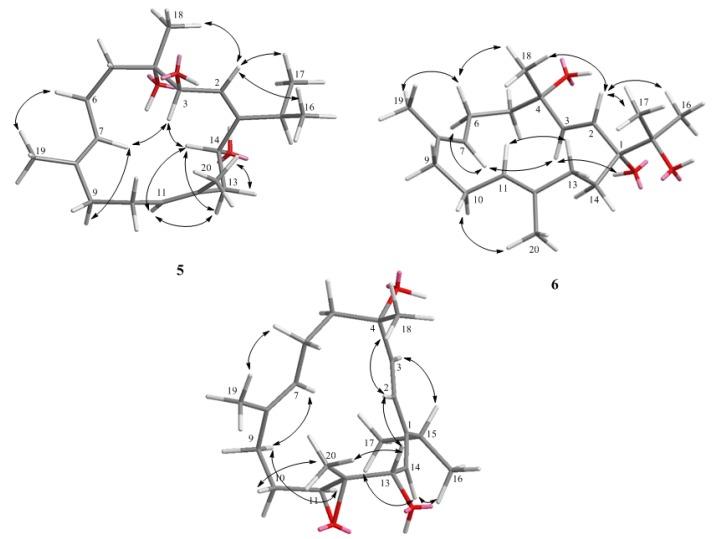
Key NOE correlations of **5**–**7**.

Klyflaccicembranol E (**5**) was obtained as a colorless oil. It possessed the molecular formula C_20_H_34_O_3_ and four unsaturations, as concluded from the pseudomolecular ion peak [M + Na]^+^ at *m*/*z* 345.2404 in the HRESIMS. The presence of hydroxy groups in **5** was demonstrated by an IR absorption band at ν_max_ 3408 cm^−1^ and ^13^C NMR signals at δ_C_ 75.2 (C), 70.7 (CH), and 70.6 (CH). The NMR spectroscopic data comparison of **5** with semisynthetic product **11** (Figure 6), obtained by chromic acid oxidation of sarcophytol A [22], suggested that **5** (C_20_H_34_O_3_) was the hydrated derivative of **11** (C_20_H_32_O_2_). Therefore, the 3,14-dihydroxy compound **5**, relative to the 3,14-epoxy compound **11**, exhibited significant upfield (∆δ_C_ − 8.5, −6.5, −13.9 ppm) and downfield (∆δ_C_ + 3.6 ppm) shifts at C-2, C-3, C-14, and C-1, respectively. Furthermore, extensive interpretation of 2D NMR correlations further established the gross structure of compound **5** to be 3,4,14-trihydroxycembra-1(2),7,11-triene (Figure 2). The geometries of the double bonds and stereochemistries at C-3, C-4, and C-14 were determined by careful investigation of NOE correlations exhibited in the NOESY spectrum (Figure 7) in combination with molecular modelling. The NOE correlations of the olefinic H-2 with protons of the isopropyl methyls (H_3_-16 and H_3_-17) revealed the *Z* geometry of the double bond at C-1/C-2. The chemical shift values of C-19 (15.1) and C-20 (17.1) reflected the *E* geometries of the trisubstituted double bonds (δ_C_ < 20 ppm) at C-7/C-8 and C-11/C-12 in the molecule of **5**. Assuming an *S*-configuration at C-4 (as found in biogenetically-related metabolites **1**, **2**, and **8**), the NOE interaction of H-2 with H_3_-18, but not with H-3, reflected the *R*-configuration at C-3. Consequently, the *S*-configuration was allocated for C-14, as H-14 exhibited strong NOE correlation with H-3. The above-mentioned findings, along with detailed analysis of other NOE correlations (Figure 7), identified klyflaccicembranol E (**5**) as (3*R*,4*S*,14*S*,1*Z*,7*E*,11*E*)-3,4,14-trihydroxycembra-1,7,11-triene.

Klyflaccicembranol F (**6**) displayed a pseudomolecular ion peak at *m*/*z*, 345.2405 ([M + Na]^+^) in the HRESIMS, consistent with a molecular formula of C_20_H_34_O_3_ and four degrees of unsaturation. Its IR spectrum also showed a broad absorption band at ν_max_ 3392 cm^−1^, representing a hydroxy group. This was further supported by NMR signals resonating at δ_C_ 80.9, 75.1, and 71.9 (each C) of tertiary hydroxyl-bearing carbons and a hydroxy proton at δ_H_ 2.60 (1H, s). Moreover, carbon signals appearing at δ_C_ 129.2 (C-2, CH), 138.0 (C-3, CH), 128.6 (C-7, CH), 132.7 (C-8, C), 126.9 (C-11, CH), and 136.1 (C-12, C) indicated the presence of one disubstituted double bond and two trisubstituted olefins in **6**, respectively. The analysis of COSY correlations (Figure 2) of **6** indicated four consecutive proton sequences. The connection of four partial structures was subsequently resolved by the HMBC. Furthermore, long-range correlations observed from both H_3_-16 and H_3_-17 (δ_H_ 1.13 and 1.21, each 3H, s) to C-15 (δ_C_ 75.1, C) and C-1 (δ_C_ 80.9, C), 1-OH (δ_H_ 2.60, 1H, s) to C-1 and C-14, and H-2 (δ_H_ 5.61, 1H, d, *J* = 16.0 Hz) to C-1 and C-4 (δ_C_ 71.9, C), enabled localization of the hydroxy groups at C-15, C-1, and C-4, respectively. The planar structure of compound **6** was thus described as 1,4,15-trihydroxycembra-2,7,11-triene (Figure 2). Comparison of NMR data of **6** with the known cembranoid crassumol A (**12**) [23] revealed that **6** had similar ^1^H and ^13^C chemical shifts, except for the significant downfield shifts noticed at C-3, C-4, C-5 (∆δ_C_ + 1.1, +1.8, and +1.5, respectively) and H-2 (∆δ_H_ + 0.18), and upfield shifts at C-2 (∆δ_C_ − 0.9 ppm) and H-3 (∆δ_H_ − 0.18 ppm), which suggested klyflaccicembranol F (**6**) to be the 1-epimer of **12** (Figure 6). Careful investigation of key NOE correlations (Figure 7) enabled us to prove the α-orientation of 1-OH. In the NOESY spectrum of **6**, H_3_-18 was found to exhibit correlation with H-2, and H-2 with both H_3_-16 and H_3_-17; therefore, assuming a β-orientation of H_3_-18, H-2 and the isopropyl methyls should also be positioned on the β-face. Therefore, 1-OH and 4-OH should be α-oriented. The chemical shift values of C-19 (δ_C_ 14.7) and C-20 (δ_C_ 14.8) reflected the *E* geometries of the trisubstituted double bonds at C-7/C-8 and C-11/C-12 in the molecule of **6**. Moreover, it should be noted that the large *J_HH_* values for H-2 and H-3 (δ_C_ 16.0 Hz) and the null NOE response reflected the *trans* positions of these two protons. On the basis of the above findings, metabolite **6** was determined to be (1*R*,4S,2*E*,7*E*,11*E*)-1,4,15- trihydroxycembra-2,7,11-triene.

New metabolite **7** was also isolated as a colorless oil, with the molecular formula C_20_H_32_O_3_, as indicated by HRESIMS (*m*/*z* 343.2250 [M + Na]^+^). Substitution of the hydroxy group of this compound was revealed by the IR absorption band at ν_max_ 3419 cm^−1^ and ^13^C NMR signals at δ_C_ 73.0 (C) and 71.6 (CH). The two proton signals resonating at δ_H_ 6.24 and 5.75 (each 1H, d, *J* = 16.0 Hz) were found to represent two olefinic protons correlated in the HSQC spectrum with the carbon signals at δ_C_ 123.7 and 138.3 (each CH), respectively, attributable to a *trans* 1,2-disubstituted double bond. Moreover, carbon signals at δ_C_ 146.0 (C), 131.9 (C), 127.6 (CH), 122.9 (CH), 64.7 (C), and 61.3 (CH) indicated the presence of two trisubstituted double bonds and a trisubstituted epoxide in **7**. The remaining ten carbons were assigned to five methyls, one *sp*^3^ methine and four methylene groups. By NMR spectroscopic data comparison, it was found that this compound was the 13-hydroxy derivative of **10**, isolated in this study and previously from *S. gibberosa* [16]. The hydroxy group at C-13 in **7** induced a significant upfield shift at H-13 (∆δ_H_ − 1.21 ppm) and a downfield shift at C-14 (∆δ_C_ + 5.0 ppm) relative to **10**. The structure of **7** was unambiguously determined by the extensive analysis of COSY and HMBC (Figure 2) and NOESY correlations (Figure 7). In addition, the appearance of the 1H double doublet signal of H-13 (δ_H_ 4.59, 1H, dd, *J* = 8.0, 8.0 Hz) was due to *vicinal* coupling with the olefinic H-14 (δ_H_ 5.10, 1H, d, *J* = 8.0 Hz) and the free proton of the hydroxy group at C-13 (δ_H_ 1.74, 1H, d, *J* = 8.0 Hz). Comparison of the splitting patterns of H-13 and H-14 of **7** (d, *J* = 8.0 Hz) with those of known cembranoid **10** (d, *J* = 9.0 Hz) suggested the same stereochemistry at C-13. The structure of klyflaccicembranol G (**7**) was thus established as (4*S*,11*S*,12*S*,13*S*,2*E*,7*E*,14*Z*)-4,13-dihydroxy-11,12-epoxy-cembra-2,7,14-triene.

Klyflaccicembranol I (**9**) possessed the molecular formula C_20_H_32_O_3_, as revealed from the ESIMS (*m*/*z* 343 [M + Na]^+^) and NMR data (Table 1 and Table 3). The ^1^H and ^13^C NMR data demonstrated the characteristic features of non-lactonized cembranoids (C_20_ signals, including those of five methyls) isolated previously from soft corals of the genus *Sinularia* and* Sarcophyton* [16,17,18,24,25]. By careful spectral comparison, it was found that the ^1^H and ^13^C NMR data of **9** were identical to those of 11,12-epoxy-13,14-dihydroxycembrene obtained by hydrolysis of flaccidoxide [26], including the magnitude and sign of optical rotation ${\left[\alpha \right]}_{D}^{25}$ +124.

Cytotoxicity of metabolites **1**–**6** and **8**–**10** against the growth of HT-29 (human colon adenocarcinoma), A549 (human lung adenocarcinoma), K562 (human erythromyeloblastoid leukemia), and P388 (mouse lymphatic leukemia) cell lines was evaluated. With the exception of inactive metabolites **1**, **3**, **5**, and **10**, all the other compounds exhibited variable potency against the tested cell lines (Table 4). Compounds **4** and **6** showed cytotoxicity against K562 and A549 (IC_50_ 44.9 and 21.4 μM, respectively). Compound **8** was capable of affecting the growth of three cancer cell lines (A549, K562, and P388) in the range of IC_50_ 34.6–49.4 μM; however, it was found to be doubly-potent (IC_50_ 49.4 μM) relative to the positive control fluorouracil (IC_50_ 110 μM) against A549 cancer cells. Compound **2** was cytotoxic against two cell lines (A549 and K562), being 6.5-fold more potent than the positive control against the growth of A549. In addition, compound **9** was cytotoxic against another pair of cancer cells (HT-29 and P338), being very potent against P388.

**Figure 8:**
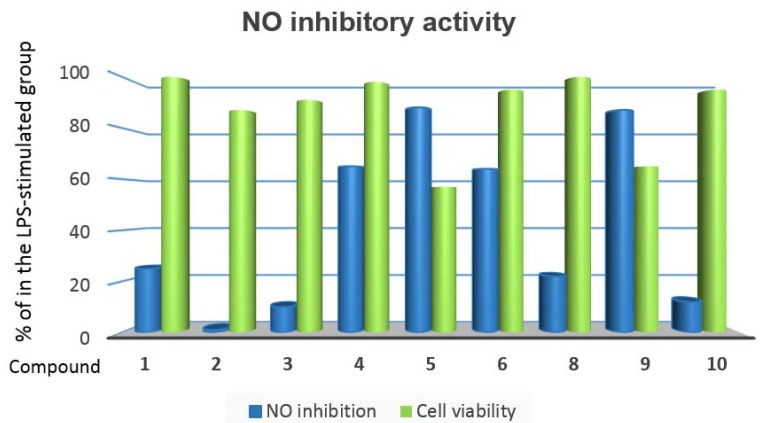
Inhibitory effects of compounds **1**–**6** and **8**–**10** at 50 μg/mL on nitric oxide (NO) production in lipopolysaccharide‎ (LPS)-stimulated RAW264.7 cells.

The isolated compounds **1**–**6** and **8**–**10** also were evaluated in terms of their ability to suppress NO in LPS-activated RAW264.7 macrophages (Figure 8). The results showed that cembranoids **5** and **9** strongly inhibited 88% and 87% of NO production at 50 μg/mL, respectively. However, compounds **4** and **6** at the same dose possessed moderate potency (65% and 64% NO inhibition), with IC_50_ values of 46.7 and 47.0 μg/mL, respectively. The higher cell viability indexes attained by **4** and **6** (98% and 95%, respectively) relative to **5** and **9** represented an advantageous characteristic in addition to the NO inhibitory effect over **5** or **9**. A positive control, curcumin, at 10 μg/mL succeeded under the same experimental conditions in reducing the NO level by 92.5% (IC_50_ 6.3 μg/mL), in association with 98% retention of cell viability. With the exception of the inactive metabolite **2**, the rest of the tested compounds (**1**, **3**, **8** and **10**) showed weak NO inhibitory activity (12%–25%).

## 3. Materials and Methods 

### 3.1. General Procedures

Optical rotations were measured on a JASCO P-1020 polarimeter (JASCO, Tokyo, Japan). IR spectra were recorded on a JASCO FT/IR-4100 spectrophotometer (JASCO). Ultraviolet spectra were recorded on a JASCO V-650 spectrophotometer. ESIMS and HRESIMS spectral data were recorded on a BRUKER APEX II mass spectrometer (Bruker, Bremen, Germany). The NMR spectra were recorded on a Varian Unity INOVA 500 FT-NMR at 500 MHz for ^1^H and 125 MHz for ^13^C or on a Varian 400 FT-NMR at 400 MHz for ^1^H and 100 MHz for ^13^C or on a Bruker AMX-300 FT-NMR at 300 MHz for ^1^H and 75 MHz for ^13^C, in CDCl_3_ or C_6_D_6_ using TMS as internal standard (δ in ppm, *J* in Hz). Silica gel 60 (Merck, 230–400 mesh), precoated silica gel plates (Merck, Darmstadt, Germany, Kieselgel 60 F254, 0.2 mm) were used for open CC and analytical TLC analysis, respectively. Isolation by HPLC was performed by a Hitachi L-2455 instrument equipped with a reversed-phase (RP-18) column (GL Sciences Inc., Tokyo, Japan ODS-3, 5 μm, 250 × 20 mm).

### 3.2. Animal Material

The soft coral *Klyxum flaccidum* Tixier-Durivault (Alcyoniidea) was collected by hand via SCUBA off the coast of Hsiao Liuchiu Island (22°19′48″ N 120°21′55″ E; Pingtung County), in October 2011, at a depth of 10–15 m along the coast of the island of Pratas, Taiwan. The species identification is based on three levels of morphological characters, i.e., colony shape, polyps’ morphology and the morphology of sclerites in different parts of the coral colony, and then stored at −20 °C until extraction. A voucher sample (specimen no. LI6) was deposited at the Department of Marine Biotechnology and Resources, National Sun Yat-sen University (Kaohsiung, Taiwan). The organism was identified by Professor Chang-Feng Dai, Institute of Oceanography, National Taiwan University, Taipei 112, Taiwan. 

### 3.3. Extraction and Separation

The frozen bodies of *K. flaccidum* (8.0 kg, wet weight) were sliced and exhaustively extracted with EtOAc and filtered off. The solvent-free residue (120 g) was fractionated by silica gel column chromatography, using EtOAc–*n*-hexane (0:100 to 100:0, gradient) and then MeOH–EtOAc (0:100 to 100:0, gradient) as eluting solvents, in order to yield 26 fractions (F1 to F26). F8 eluted with EtOAc–*n*-hexane (1:2) was further isolated on silica gel, using EtOAc–*n*-hexane (1:3 to 2:3, stepwise) to yield **3** (2.5 mg), **2** (1.5 mg), and **8** (40.5 mg), respectively. F10 eluted with EtOAc–*n*-hexane (1:1) was separated on silica gel, using EtOAc–*n*-hexane (1:2) to give four subfractions F101–F104. F101 was isolated on RP-HPLC using MeOH–H_2_O (3:1) to give **4** (10.2 mg), **9** (9.7 mg), and **10** (11.3 mg), respectively. F12 eluted with EtOAc–*n*-hexane (2:1) was re-chromatographed on silica gel column, using EtOAc–*n*-hexane (1:2–2:1), in order to give four subfractions F121–F124. F122 was isolated on RP-HPLC, using MeOH–H_2_O (15:1) as a mobile phase, to yield **1** (1.8 mg), **5** (3.4 mg), **6** (2.1 mg), and **7** (3.2 mg). 

#### 3.3.1. Klyflaccicembranol A (**1**)

Colorless oil; ${\left[\alpha \right]}_{D}^{25}$ +57.2 (*c* 0.5, CHCl_3_); IR (neat) ν_max_ 3445, 2959, 2924, 2870, 1456, 1381, 1118, 1087, 1064, 850 and 738 cm^−1^; ^13^C (100 MHz, C_6_D_6_) and ^1^H NMR (400 MHz, C_6_D_6_) data, see Table 1 and Table 2, respectively; ESIMS *m*/*z* 359 [M + Na]^+^; HRESIMS *m*/*z* 359.2196 [M + Na]^+^ (calcd. for C_20_H_32_O_4_Na, 359.2198).

#### 3.3.2. Klyflaccicembranol B (**2**)

Colorless oil; ${\left[\alpha \right]}_{D}^{25}$ +65.9 (*c* 0.4, CHCl_3_); IR (neat) ν_max_ 3443, 2924, 2856, 1746, 1457, 1375, and 1234 cm^−1^; ^13^C (125 MHz, CDCl_3_) and ^1^H NMR (500 MHz, CDCl_3_) data, see Table 1 and Table 2, respectively; ESIMS *m*/*z* 417 [M + Na]^+^; HRESIMS *m*/*z* 417.2250 [M + Na]^+^ (calcd. for C_22_H_34_O_6_Na, 417.2253).

#### 3.3.3. Klyflaccicembranol C (**3**)

Colorless oil; ${\left[\alpha \right]}_{D}^{25}$ +38.9 (*c* 0.7, CHCl_3_); IR (neat) ν_max_ 3450, 2927, 2858, 1731, 1455, 1373, 1240 and 1016 cm^−1^; ^13^C (100 MHz, CDCl_3_) and ^1^H NMR (400 MHz, CDCl_3_) data, see Table 1 and Table 2, respectively; ESIMS *m*/*z* 385 [M + Na]^+^; HRESIMS *m*/*z* 385.2354 [M + Na]^+^ (calcd. for C_22_H_34_O_4_Na, 358.2355).

#### 3.3.4. Klyflaccicembranol D (**4**)

Pale yellow oil; ${\left[\alpha \right]}_{D}^{25}$ −1.51 (*c* 3.17, CHCl_3_); IR (neat) ν_max_ 3419, 2953, 2925, 2867, 1456, 1375, 977, and 757 cm^−1^; ^13^C (100 MHz, C_6_D_6_) and ^1^H NMR (400 MHz, C_6_D_6_) data, see Table 1 and Table 2, respectively; ESIMS *m*/*z* 327 [M + Na]^+^; HRESIMS *m*/*z* 327.2200 [M + Na]^+^ (calcd. for C_20_H_32_O_2_Na, 327.2198).

#### 3.3.5. Klyflaccicembranol E (**5**)

Colorless oil; ${\left[\alpha \right]}_{D}^{25}$ −70.5 (*c* 1.0, CHCl_3_); IR (neat) ν_max_ 3408, 2956, 2924, 2869, 1455, 1381, 1002, and 757 cm^−1^; ^13^C (100 MHz, CDCl_3_) and ^1^H NMR (400 MHz, CDCl_3_) data, see Table 1 and Table 2, respectively; ESIMS *m*/*z* 345 [M + Na]^+^; HRESIMS *m*/*z* 345.2404 [M + Na]^+^ (calcd. for C_20_H_34_O_3_Na, 345.2406).

#### 3.3.6. Klyflaccicembranol F (**6**)

Colorless oil; ${\left[\alpha \right]}_{D}^{25}$ −32.0 (*c* 0.6, CHCl_3_); IR (neat) ν_max_ 3392, 2924, 2855, 1455, and 1370 cm^−1^; ^13^C (125 MHz, CDCl_3_) and ^1^H NMR (500 MHz, CDCl_3_) data, see Table 1 and Table 3; ESIMS *m*/*z* 345 [M + Na]^+^; HRESIMS *m*/*z* 345.2405 [M + Na]^+^ (calcd. for C_20_H_34_O_3_Na, 345.2406).

#### 3.3.7. Klyflaccicembranol G (**7**)

Colorless oil; ${\left[\alpha \right]}_{D}^{25}$ +18.0 (*c* 1.0, CHCl_3_); IR (neat) ν_max_ 3419, 2953, 2925, 2867, 1460, 1375, 977 and 757 cm^−1^; ^13^C (100 MHz, CDCl_3_) and ^1^H NMR (400 MHz, CDCl_3_) data, see Table 1 and Table 3, respectively; ESIMS *m*/*z* 343 [M + Na]^+^; HRESIMS *m*/*z* 343.2250 [M + Na]^+^ (calcd. for C_20_H_32_O_3_Na, 343.2249).

#### 3.3.8. Klyflaccicembranol H (**8**)

Colorless oil; ${\left[\alpha \right]}_{D}^{25}$ +83.0 (*c* 1.4, CHCl_3_); IR (neat) ν_max_ 3310, 3070, 2964, 2958, 2931, 2867, 1730, 1644, 1460, 1441, 1371, 1251 and 1182 cm^−1^; ^13^C (100 MHz, CDCl_3_) and ^1^H NMR (400 MHz, CDCl_3_) data, see Table 1 and Table 3, respectively; ^1^H NMR (CD_3_OD, 500 MHz) δ_H_ 5.65 (1H, d, *J* = 2.0 Hz, H-2), 5.37 (1H, dd, *J* = 5.5, 5.5 Hz, H-7), 5.15 (1H, d, *J* = 2.0 Hz, H-13), 5.07 (1H, ddd, *J* = 5.0, 2.0, 2.0 Hz, H-14), 4.57 (1H, d, *J* = 5.0 Hz, H-3), 2.95 (1H, dd,* J* = 7.5, 1.5 Hz, H-11), 2.21 (2H, m, H_2_-9), 2.19 (1H, m, H-6), 2.13 (1H, m, H-15), 2.10 (1H, m, H-6), 1.93 (3H, s, Ac), 1.87 (1H, dd, *J* = 14.0, 9.5 Hz, H-5), 1.81 (1H, m, H-10), 1.70 (1H, m, H-10), 1.64 (1H, dd, *J* = 14.0, 8.0 Hz, H-5), 1.60 (3H, s, H_3_-19), 1.39 (3H, s, H_3_-20), 1.10 (3H, dd, *J* = 7.0 Hz, H_3_-17), 1.06 (3H, dd, *J* = 7.0 Hz, H_3_-16), 0.96 (3H, s, H_3_-18); ^13^C NMR (CD_3_OD, 125 MHz) δ_C_ 171.7 (C, 13-OAc), 148.4 (C, C-1), 133.2 (C, C-8), 128.2 (CH, C-7), 122.9 (CH, C-2), 90.6 (CH, C-3), 86.2 (CH, C-14), 75.2 (C, C-4), 74.2 (CH, C-13), 62.3 (C, C-12), 61.0 (CH, C-11), 42.1 (CH_2_, C-5), 37.7 (CH_2_, C-9), 25.6 (CH_2_, C-10), 22.7 (CH_2_, C-6), 25.7 (CH, C-15), 22.7 (CH_3_, C-6), 22.7 (CH_3_, C-18), 22.6 (CH_3_, C-16), 21.3 (CH_3_, C-17), 20.6 (CH_3_, 13-OAc), 16.8 (CH_3_, C-19), 16.2 (CH_3_, C-20); ESIMS *m*/*z* 401 [M + Na]^+^, 385 [M − O + Na]^+^, 325 [M − AcOH − O + Na]^+^; EIMS *m*/*z* 379 [M + H]^+^, 361 [M − H_2_O]^+^, 318 [M − AcOH]^+^, 300 [M − AcOH − H_2_O]^+^; HRESIMS *m*/*z* 378.2404 [M]^+^ (calcd. for C_22_H_34_O_5_, 378.2406).

#### 3.3.9. Klyflaccicembranol I (**9**)

Colorless oil; ${\left[\alpha \right]}_{D}^{25}$ +124.0 (*c* 2.7, CHCl_3_); IR (neat) ν_max_ 3487, 3275, 2955, 2933, 2868, 1732, 1644, 1453, 1370, 1247 and 1183 cm^−1^; ^13^C (100 MHz, C_6_D_6_) and ^1^H NMR (400 MHz, C_6_D_6_) data, see Table 1 and Table 3, respectively; ESIMS *m*/*z* 343 [M + Na]^+^.

#### 3.3.10. Hydrolysis of Klyflaccicembranol H (**8**)

A solution of **8** (3.2 mg, 8.12 μmol) in MeOH (2.0 mL) was stirred with K_2_CO_3_ (30 mg) overnight at room temperature. The reaction mixture was diluted with distilled water (3.0 mL) and followed by extraction with CH_2_Cl_2_. The organic extract was then purified on short silica gel CC, using 25% EtOAc in *n*-hexane as an eluting solvent, to afford **1** (2.4 mg, 6.82 μmol, 84% yield). **1**: colorless oil; ^1^H NMR (CDCl_3_, 300 MHz). δ_H_ 5.64 (1H, br s, H-2), 5.32 (1H, dd, *J* = 5.5, 5.5 Hz, H-7), 3.81 (1H, d, *J* = 1.8 Hz, H-13), 4.92 (1H, br d, *J* = 5.5 Hz, H-14), 4.57 (1H, d, *J* = 5.5 Hz, H-3), 3.12 (1H, dd,* J* = 9.5, 3.6 Hz, H-11), 2.20 (2H, m, H_2_-9), 2.13 (2H, m, H_2_-6), 2.37 (1H, septet, *J* = 6.9 Hz, H-15), 1.89 (1H, m, H-5a), 1.83 (2H, m, H_2_-10), 1.65 (1H, m, H-5b), 1.59 (3H, s, H_3_-19), 1.36 (3H, s, H_3_-20), 1.10 (3H, dd, *J* = 6.9 Hz, H_3_-17), 1.08 (3H, dd, *J* = 6.9 Hz, H_3_-16), 1.01 (3H, s, H_3_-18); ^13^C NMR (CDCl_3_, 75 MHz) δ_C_ 148.4 (C, C-1), 132.8 (C, C-8), 126.8 (CH, C-7), 122.9 (CH, C-2), 89.6 (CH, C-3), 85.6 (CH, C-14), 74.9 (C, C-4), 72.6 (CH, C-13), 62.4 (C, C-12), 60.3 (CH, C-11), 41.6 (CH_2_, C-5), 36.9 (CH_2_, C-9), 24.8 (CH_2_, C-10), 22.1 (CH_2_, C-6), 26.6 (CH, C-15), 22.1 (CH_3_, C-6), 22.7 (CH_3_, C-18), 22.4 (CH_3_, C-16), 21.4 (CH_3_, C-17), 16.9 (CH_3_, C-19), 15.7 (CH_3_, C-20).

#### 3.3.11. Preparation of (*S*)- and (*R*)-MTPA Esters of **1**

To a solution of **1** (1.1 mg, 3.1 μmol) in pyridine (50 μL), *R*-(−)-MTPA chloride (5 μL) was added and allowed to react overnight at room temperature. The reaction was terminated by the addition of 1.0 mL of water, and then processed as previously described [27] to yield the (*S*)-MTPA ester **1a** (1.2 mg, 2.1 μmol, 67.7%). Similarly, the correspondent (*R*) -MTPA ester **1b** was also obtained from the reaction of *S*-(+)-MTPA chloride with **1**. ^1^H NMR (CDCl_3_, 300 MHz) of **1a**: δ_H_ 5.496 (1H, br s, H-2), 5.341 (1H, br s, H-13), 5.185 (1H, dd, *J* = 4.5, 4.5 Hz, H-7), 5.030 (1H, br d, *J* = 4.5 Hz, H-14), 4.324 (1H, br d, *J* = 4.5 Hz, H-3), 2.609 (1H, m, H-11), 2.177 (1H, m, H-15), 2.100 (4H, m, H_2_-6 and H_2_-9), 1.985 (1H, m, H-5α), 1.825 (1H, m, H-5β), 1.666 (2H, m, H_2_-10), 1.536 (3H, s, H_3_-19), 1.387 (3H, s, H_3_-20), 1.097 (3H, d, *J* = 6.9 Hz, H_3_-17), 1.040 (3H, d,* J* = 6.9 Hz, H_3_-16), 0.962 (3H, s, H_3_-18). ^1^H NMR (CDCl_3_, 300 MHz) of **1b**: δ_H_ 5.639 (1H, br s, H-2), 5.347 (1H, br s, H-13), 5.223 (1H, dd,* J* = 4.5, 4.5 Hz, H-7), 5.075 (1H, br d, *J* = 4.5 Hz, H-14), 4.542 (1H, br d, *J* = 4.5 Hz, H-3), 2.623 (1H, m, H-11), 2.176 (1H, m, H-15), 2.111 (4H, m, H_2_-6 and H_2_-9), 1.994 (1H, m, H-5α), 1.858 (1H, m, H-5β), 1.637 (2H, m, H_2_-10), 1.543 (3H, s, H_3_-19), 1.382 (3H, s, H_3_-20), 1.122 (3H, d, *J* = 6.9 Hz, H_3_-17), 1.057 (3H, d, *J* = 6.9 Hz, H_3_-16), 1.003 (3H, s, H_3_-18).

### 3.4. Cytotoxicity Assay

Cancer cell (HT-29, A549, K562, and P388) lines were purchased from the American Type Culture Collection (ATCC). Evaluation of cytotoxicity for the isolated metabolites from *K. flaccidum* was performed according to Alamar Blue assay [14,15].

### 3.5. Nitric Oxide Inhibitory Assay

The inhibitory activity of isolated compounds on nitric oxide (NO) production by murine RAW 264.7 macrophage cells was assessed according to Griess reaction. Briefly, cells were cultured in 96-well plates for 1 h. The cells were challenged with LPS (5 μg/mL) and test samples for 48 h. The culture supernatant (100 μL) were reacted then with Griess reagent (1:1 mixture of 0.1% *N*-(1-naphthyl) ethylene-diamine dihydrochloride in water and 1% sulfanilamide in 5% phosphoric acid, 100 μL) in a 96-well plate, and absorbance was measured using the ELISA reader at 540 nm [28,29].

## 4. Conclusions

For the first time, cembranoid-based compounds were isolated and identified from the soft corals of genus *Klyxum* by this study. Five metabolites (klyflaccicembranols B, D, F, H, and I) exhibited variable activities against a limited panel of cancer cell lines while klyflaccicembranols D–F and I showed strong anti-inflammatory effect through inhibition of NO production in LPS-stimulated RAW264.7 macrophages.

## Figures and Tables

**Table 1:** ^13^C NMR spectral data of compounds **1**–**9**.

#	1 *^a^*	2 *^b^*	3 *^c^*	4 *^a^*	5 *^c^*	6 *^b^*	7 *^c^*	8 *^c^*	9 *^a^*
1	147.8 (C)	147.7 (C)	155.9 (C)	146.3 (C)	154.6 (C)	80.9 (C)	146.0 (C)	147.4 (C)	146.9 (C)
2	121.0 (CH) *^d^*	121.2 (CH)	67.2 (CH)	124.3 (CH)	122.7 (CH)	129.2 (CH)	123.7 (CH)	121.2 (CH)	120.8 (CH)
3	89.7 (CH)	91.1 (CH)	126.3 (CH)	136.2 (CH)	70.7 (CH)	138.0 (CH)	138.3 (CH)	89.1 (CH)	121.0 (CH)
4	74.2 (C)	74.6 (C)	137.6 (C)	72.9 (C)	75.2 (C)	71.9 (C)	73.0 (C)	74.6 (C)	136.6 (C)
5	41.6 (CH_2_)	40.5 (CH_2_)	39.0 (CH_2_)	45.5 (CH_2_)	38.6 (CH_2_)	43.8 (CH_2_)	39.0 (CH_2_)	41.3 (CH_2_)	39.1 (CH_2_)
6	22.1 (CH_2_)	25.2 (CH_2_)	24.2 (CH_2_)	121.3 (CH)	22.3 (CH_2_)	22.3 (CH_2_)	24.2 (CH_2_)	21.8 (CH_2_)	25.6 (CH_2_)
7	127.0 (CH)	64.6 (CH)	125.2 (CH)	141.4 (CH)	127.0 (CH)	128.6 (CH)	127.6 (CH)	126.3 (CH)	126.5 (CH)
8	132.1 (C)	59.9 (C)	133.2 (C)	72.5 (C)	133.9 (C)	132.7 (C)	131.9 (C)	132.6 (C)	133.8 (C)
9	36.8 (CH_2_)	36.7 (CH_2_)	36.9 (CH_2_)	43.6 (CH_2_)	39.0 (CH_2_)	39.0 (CH_2_)	36.7 (CH_2_)	36.8 (CH_2_)	36.6 (CH_2_)
10	25.1 (CH_2_)	23.6 (CH_2_)	24.3 (CH_2_)	23.5 (CH_2_)	24.2 (CH_2_)	23.8 (CH_2_)	24.3 (CH_2_)	24.5 (CH_2_)	24.8 (CH_2_)
11	59.7 (CH)	59.0 (CH)	59.7 (CH)	126.7 (CH)	125.6 (CH)	126.9 (CH)	61.3 (CH)	59.2 (CH)	59.9 (CH)
12	61.9 (C)	60.3 (C)	61.3 (C)	132.8 (C)	131.9 (C)	136.1 (C)	64.7 (C)	60.4 (C)	62.9 (C)
13	72.8 (CH)	71.9 (CH)	71.6 (CH)	37.3 (CH_2_)	44.1 (CH_2_)	36.1 (CH_2_)	71.6 (CH)	72.7 (CH)	75.2 (CH)
14	85.8 (CH)	85.0 (CH)	115.6 (CH)	122.0 (CH)	70.6 (CH)	29.9 (CH_2_)	122.9 (CH)	84.9 (CH)	68.1 (CH)
15	26.6 (CH)	25.7 (CH)	27.6 (CH)	31.6 (CH)	27.8 (CH)	75.1 (C)	32.1 (CH)	25.7 (CH)	28.2 (CH)
16	22.2 (CH_3_)	20.9 (CH_3_)	23.7 (CH_3_)	22.6 (CH_3_)	22.8 (CH_3_)	24.5 (CH_3_)	22.1 (CH_3_)	22.2 (CH_3_)	24.0 (CH_3_)
17	21.2 (CH_3_)	20.6 (CH_3_)	24.5 (CH_3_)	22.6 (CH_3_)	23.5 (CH_3_)	24.7 (CH_3_)	22.3 (CH_3_)	20.9 (CH_3_)	25.4 (CH_3_)
18	23.1 (CH_3_)	22.2 (CH_3_)	15.7 (CH_3_)	29.7 (CH_3_)	25.1 (CH_3_)	27.8 (CH_3_)	30.0 (CH_3_)	22.6 (CH_3_)	16.7 (CH_3_)
19	16.7 (CH_3_)	16.8 (CH_3_)	15.2 (CH_3_)	29.6 (CH_3_)	15.1 (CH_3_)	14.7 (CH_3_)	15.1 (CH_3_)	16.7 (CH_3_)	15.2 (CH_3_)
20	15.5 (CH_3_)	16.0 (CH_3_)	15.2 (CH_3_)	17.6 (CH_3_)	17.1 (CH_3_)	14.8 (CH_3_)	15.5 (CH_3_)	16.0 (CH_3_)	16.1 (CH_3_)
OAc		169.9 (C)	170.7 (C)					169.9 (C)	
		21.0 (CH_3_)	21.1 (CH_3_)					20.6 (CH_3_)	

Spectra recorded in *^a^* C_6_D_6_ at 100 MHz; *^b^* CDCl_3_ at 125 MHz; and *^c^* CDCl_3_ at 100 MHz at 25 °C; *^d^* Attached protons were determined by distortionless enhancement by polarization transfer (DEPT) experiments. Values are presented as ppm downfield from tetramethylsilane (TMS).

**Table 2:** ^1^H NMR spectral data for compounds **1**–**5**.

#	1 *^a^*	2 *^b^*	3 *^c^*	4 *^a^*	5 *^c^*
2	5.59 br s	5.65 br s	5.70 d (10.0)	6.19 d (16.4)	5.41 d (7.6)
3	4.61 d (4.8) *^d^*	4.78 d (5.0)	5.25 d (10.0)	5.83 d (16.4)	4.34 d (7.6)
5	1.48 m; 1.85, m	1.82 m; 1.92 m	2.10 m; 2.24 m	2.31 2H, d (6.8)	1.55 m; 1.86 m
6	2.02 m; 2.15, m	1.59 m; 1.86 m	2.10 m; 2.27 m	5.56 dd (15.6, 6.8)	2.11 m; 2.34 m
7	5.38 dd (5.2, 5.2)	3.07 dd (6.0, 2.5)	4.83 br d (6.0)	5.52 d (15.6)	4.99 dd (6.0, 6.0)
9	2.04 m; 2.09 m	2.10 m; 1.39 m	2.16 m; 2.20 m	1.58 m; 1.67 m	1.98 m; 2.15 m
10	1.76 m; 1.83 m	1.54 m; 1.95 m	1.61 m; 1.86 m	2.01 m; 2.37 m	2.14 m; 2.18 m
11	3.15 dd (6.4, 2.0)	2.98 d (7.5)	2.47 dd (7.2, 2.0)	5.11 dd (7.2, 7.2)	4.93 dd (6.8, 6.0)
13	3.58 br s	5.21 s	5.52 d (10.4)	2.71 2H, d (8.0)	2.27 m; 2.37 m
14	4.78 br d (4.8)	5.05 d (5.0)	5.03 d (10.4)	5.48 dd (8.0, 5.6)	4.78 dd (5.6, 5.6)
15	2.25 sept (6.8)	2.17 m	2.78 sept (6.8)	2.53 sept (6.8)	2.48 m
16	0.94 3H, d (6.8)	1.05 3H, d (6.5)	1.05 3H, d (6.8)	1.09 3H, d (6.8)	1.06 3H, d (6.8)
17	1.12 3H, d (6.8)	1.10 3H, d (6.5)	1.09 3H, d (6.8)	1.10 3H, d (6.8)	1.12 3H, d (6.8)
18	0.98 3H, s	1.05 3H, s	1.80 3H, s	1.27 3H, s	1.14 3H, s
19	1.48 3H, s	1.24 3H, s	1.55 3H, s	1.16 3H, s	1.55 3H, s
20	1.18 3H, s	1.42 3H, s	1.22 3H, s	1.60 3H, s	1.68 3H, s
OAc		1.91 3H, s	2.10 3H, s		

Spectra recorded in *^a^* C_6_D_6_, at 400 MHz; *^b^* CDCl_3_ at 500 MHz; and *^c^* CDCl_3_ at 400 MHz at 25 °C; *^d^* *J* values (Hz) in parentheses.

**Table 3:** ^1^H NMR spectral data for compounds **6**–**9**.

#	6 *^a^*	7 *^b^*	8 *^b^*	9 *^c^*
2	5.61 d (16.0) *^d^*	6.24 d (16.0)	5.60 br s	6.25 d (11.2)
3	6.10 d (16.0)	5.75 d (16.0)	4.57 d (5.2)	5.85 d (11.2)
5	1.51 m; 2.01 m	2.10 m; 2.24 m	1.63 m; 1.89 m	2.00 2H, m
6	2.22 m; 2.39 m	2.10 m; 2.27 m	2.16 m; 2.18 m	1.97 m, 2.06 m
7	5.34 dd (7.5, 7.5)	5.03 dd (6.0, 6.0)	5.36 dd (5.5, 5.5)	5.03 dd (6.0, 6.0)
9	1.95 m; 2.20 m	2.16 m; 2.20 m	2.04 m; 2.09 m	1.95 m, 2.11 m
10	2.07 m; 2.24 m	1.61 m; 1.86 m	1.76 m; 1.83 m	1.43 2H, m
11	5.19 br d (9.0)	2.67 m	2.89 dd (6.0, 3.6)	3.08 dd (6.0, 6.0)
13	2.13 m; 2.19 m	4.59 dd (8.0, 8.0)	5.16 d (2.0)	3.72 d (6.0)
14	1.63 m; 2.11 m	5.10 d (8.0)	5.00 dd (5.2, 2.0)	4.60 d (6.0)
15		2.52 m	2.13 m	2.76 m
16	1.21 3H, s	1.04 3H, d (6.8)	1.03 3H, d (6.8)	1.06 d (6.8)
17	1.13 3H, s	1.07 3H, d (6.8)	1.08 3H, d (6.8)	1.28 d (6.8)
18	1.40 3H, s	1.35 3H, s	1.00 3H, s	1.59 3H, s
19	1.62 3H, s	1.60 3H, s	1.59 3H, s	1.32 3H, s
20	1.67 3H, s	1.42 3H, s	1.39 3H, s	1.34 3H, s
15-OH	2.60 s			
13-OH		1.74 d (8.0)		
OAc			2.06 3H,s	

Spectra recorded in *^a^* CDCl_3_ at 500 MHz; *^b^* CDCl_3_ at 400 MHz; and *^c^* C_6_D_6_ at 400 MHz at 25 °C; *^d^* *J* values (Hz) in parentheses.

**Table 4:** Cytotoxicities (IC_50_ μM) of compounds **1**–**6** and **8**–**10**.

Compound	HT-29	A549	K562	P388
**1**	–*^a^*	–*^a^*	–*^a^*	–*^a^*
**2**	–*^a^*	16.5	34.6	–*^a^*
**3**	–*^a^*	–*^a^*	–*^a^*	–*^a^*
**4**	–*^a^*	–*^a^*	44.9	–*^a^*
**5**	–*^a^*	–*^a^*	–*^a^*	–*^a^*
**6**	–*^a^*	21.4	–*^a^*	–*^a^*
**8**	–*^a^*	49.4	47.4	34.6
**9**	41.9	–*^a^*	–*^a^*	25.9
**10**	–*^a^*	–*^a^*	–*^a^*	–*^a^*
Fluorouracil	8.5	110	31.5	5.5

*^a^* –: Compound was considered inactive when IC_50_ > 50 μM.

## Supplementary Materials

HRESIMS, ^1^H, and ^13^C spectra of new compounds **1**–**9** are available online at www.mdpi.com/1660-3397/15/1/23/s1. Figure S1: HRESIMS spectrum of **1**, Figure S2: ^1^H NMR spectrum of **1** in C_6_D_6_ at 400 MHz, Figure S3: ^13^C NMR spectrum of **1** in C_6_D_6_ at 100 MHz, Figure S4: HRESIMS spectrum of **2**, Figure S5: ^1^H NMR spectrum of **2** in CDCl_3_ at 500 MHz, Figure S6: ^13^C NMR spectrum of **2** in CDCl_3_ at 125 MHz, Figure S7: HRESIMS spectrum of **3**, Figure S8: ^1^H NMR spectrum of **3** in CDCl_3_ at 400 MHz, Figure S9: ^13^C NMR spectrum of **3** in CDCl_3_ at 100 MHz, Figure S10: HRESIMS spectrum of **4**, Figure S11: ^1^H NMR spectrum of **4** in C_6_D_6_ at 400 MHz, Figure S12: ^13^C NMR spectrum of **4** in C_6_D_6_ at 100 MHz, Figure S13: HRESIMS spectrum of **5**, Figure S14: ^1^H NMR spectrum of **5** in CDCl_3_ at 400 MHz, Figure S15: ^13^C NMR spectrum of **5** in CDCl_3_ at 100 MHz, Figure S16: HRESIMS spectrum of **6**, Figure S17: ^1^H NMR spectrum of **6** in CDCl_3_ at 500 MHz, Figure S18: ^13^C NMR spectrum of **6** in CDCl_3_ at 125 MHz, Figure S19: HRESIMS spectrum of **7**, Figure S20: ^1^H NMR spectrum of **7** in CDCl_3_ at 400 MHz, Figure S21: ^13^C NMR spectrum of **7** in CDCl_3_ at 100 MHz, Figure S22: HRESIMS spectrum of **8**, Figure S23: ^1^H NMR spectrum of **8** in CDCl_3_ at 400 MHz, Figure S24: ^13^C NMR spectrum of **8** in CDCl_3_ at 100 MHz, Figure S25: ESIMS spectrum of **9**, Figure S26: ^1^H NMR spectrum of **9** in C_6_D_6_ at 400 MHz, Figure S27: ^13^C NMR spectrum of **9** in C_6_D_6_ at 100 MHz.
