# Supplementary material for: Klyflaccicembranols A–I, New Cembranoids from the Soft Coral Klyxum flaccidum
Source: Mar Drugs. 2017 Jan 21;15(1):23. doi: 10.3390/md15010023 (PMC5295243; doi:10.3390/md15010023)
Supplement: Supplementary file 1 [file marinedrugs-15-00023-s001.pdf]

Supplementary Materials: Klyflaccicembranols A–I, New Cembranoids from the Soft Coral *Klyxum flaccidum*

Atallah F. Ahmed, Chia-Ruei Tsai, Chiung-Yao Haung, Sheng-Yang Wang and Jyh-Horng Sheu

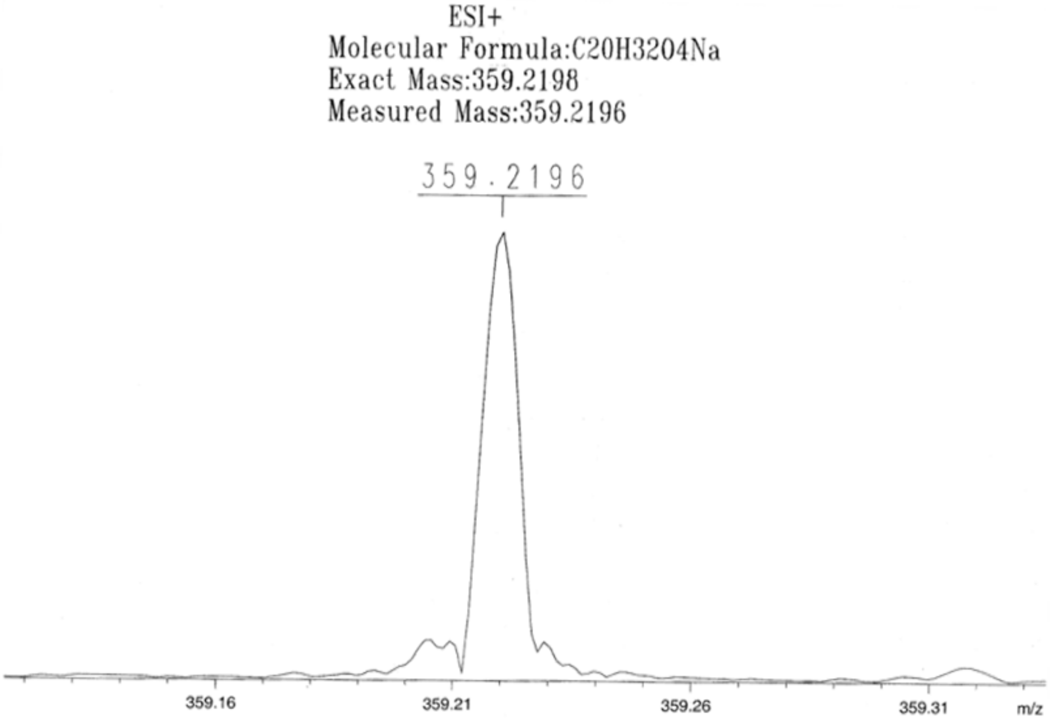

Figure S1. HRESIMS spectrum of **1**.

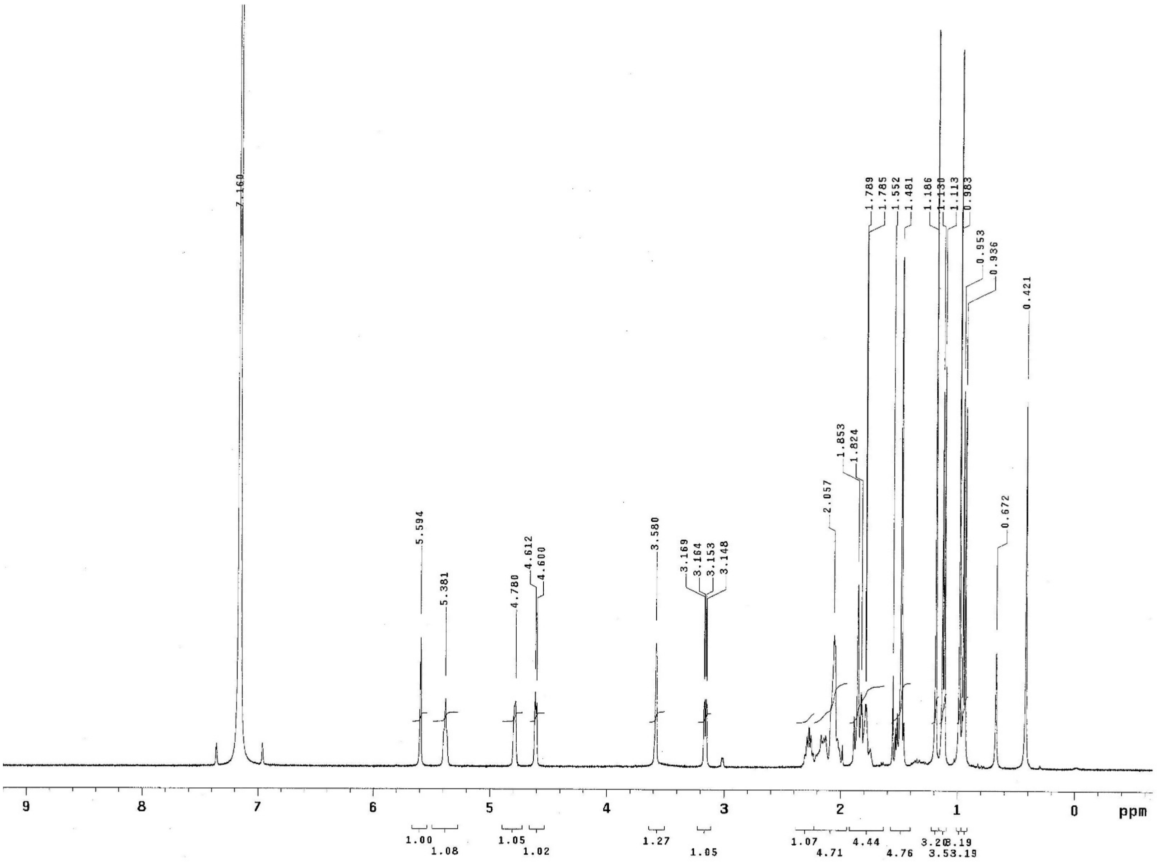

Figure S2. ^{1}H NMR spectrum of **1** in C_6D_6 at 400 MHz.

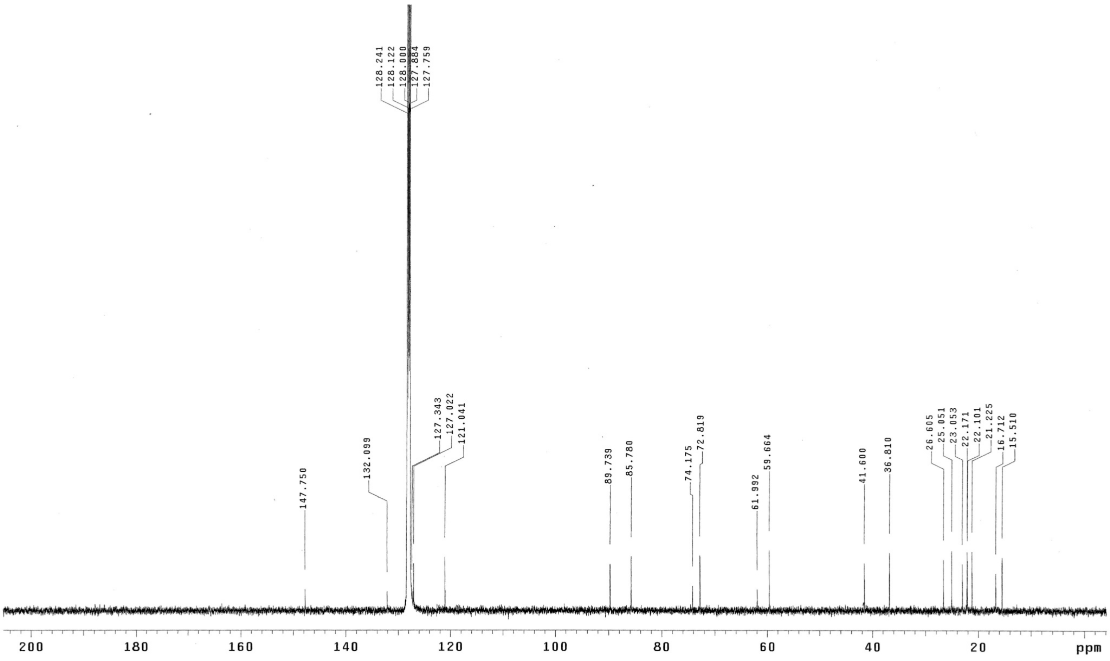

Figure S3. ^{13}C NMR spectrum of **1** in C_6D_6 at 100 MHz.

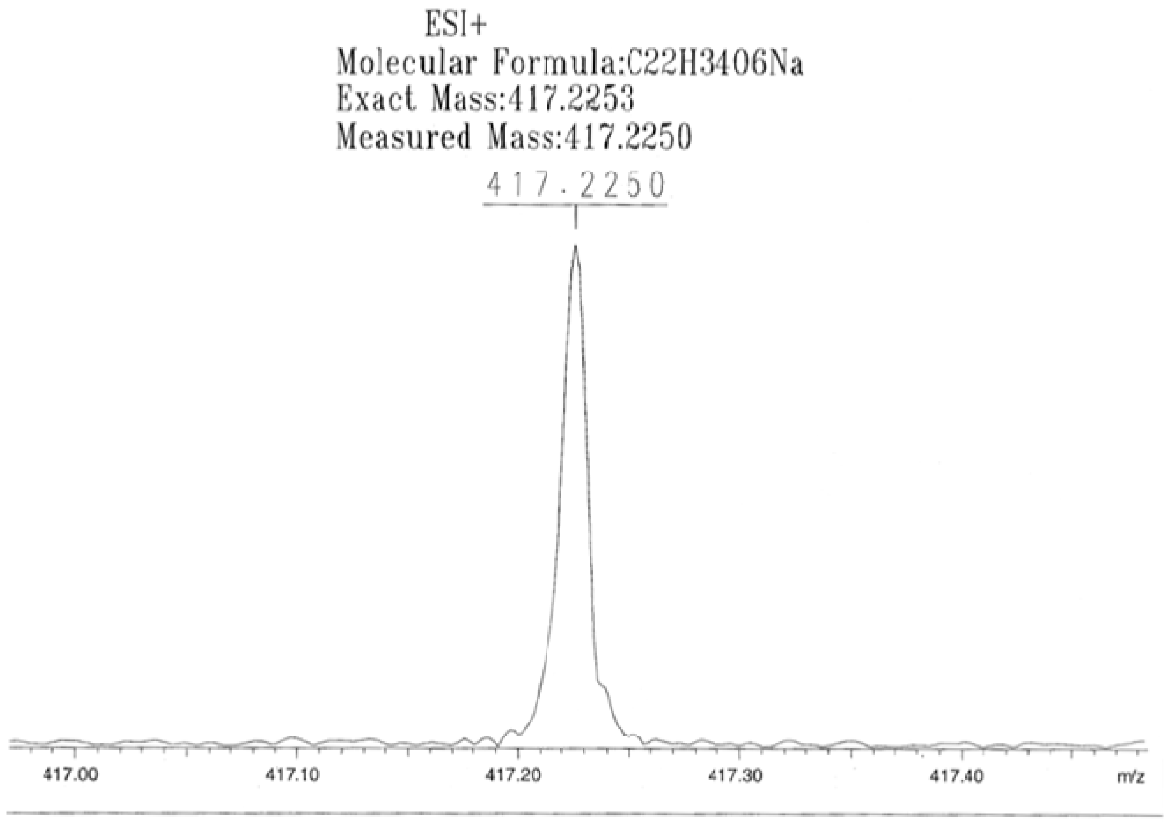

Figure S4. HRESIMS spectrum of **2**.

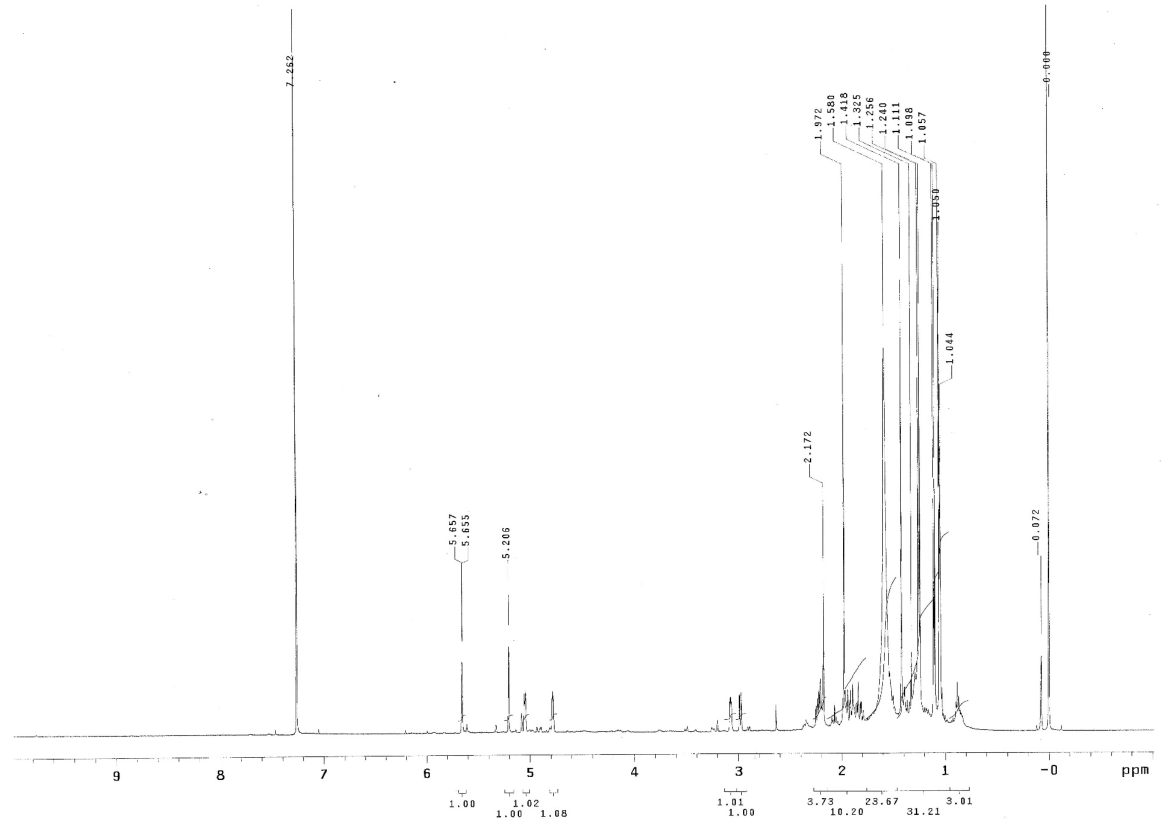

Figure S5. ^{1}H NMR spectrum of **2** in $CDCl_3$ at 500 MHz.

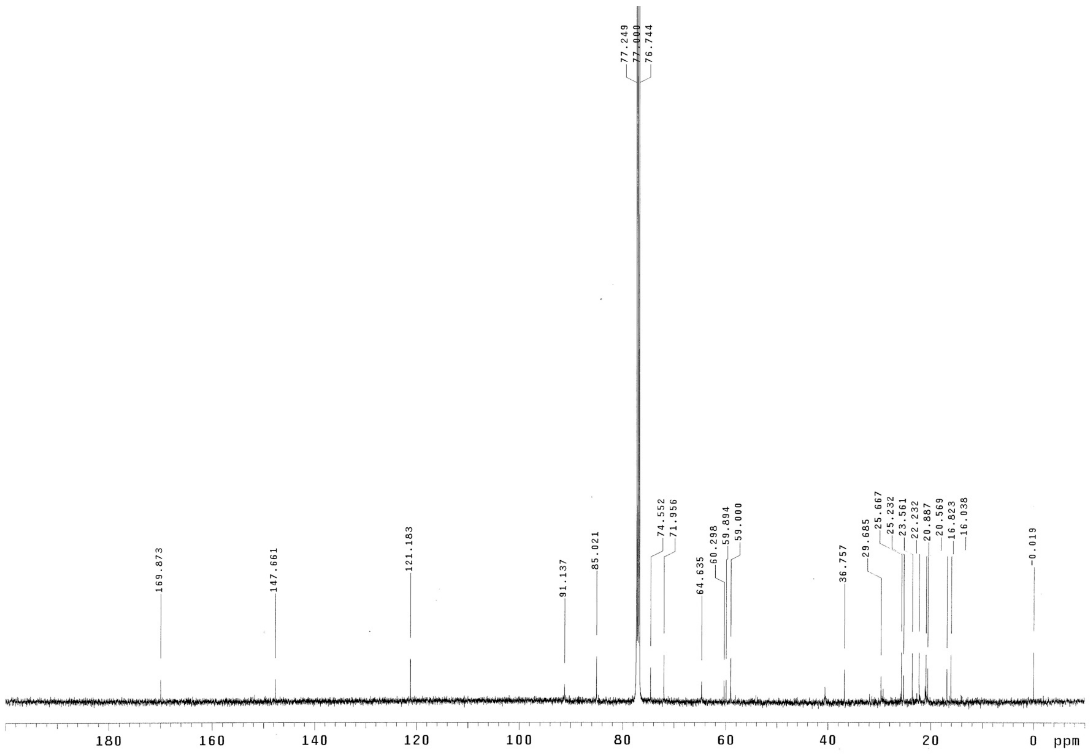

Figure S6. ^{13}C NMR spectrum of **2** in $CDCl_3$ at 125 MHz.

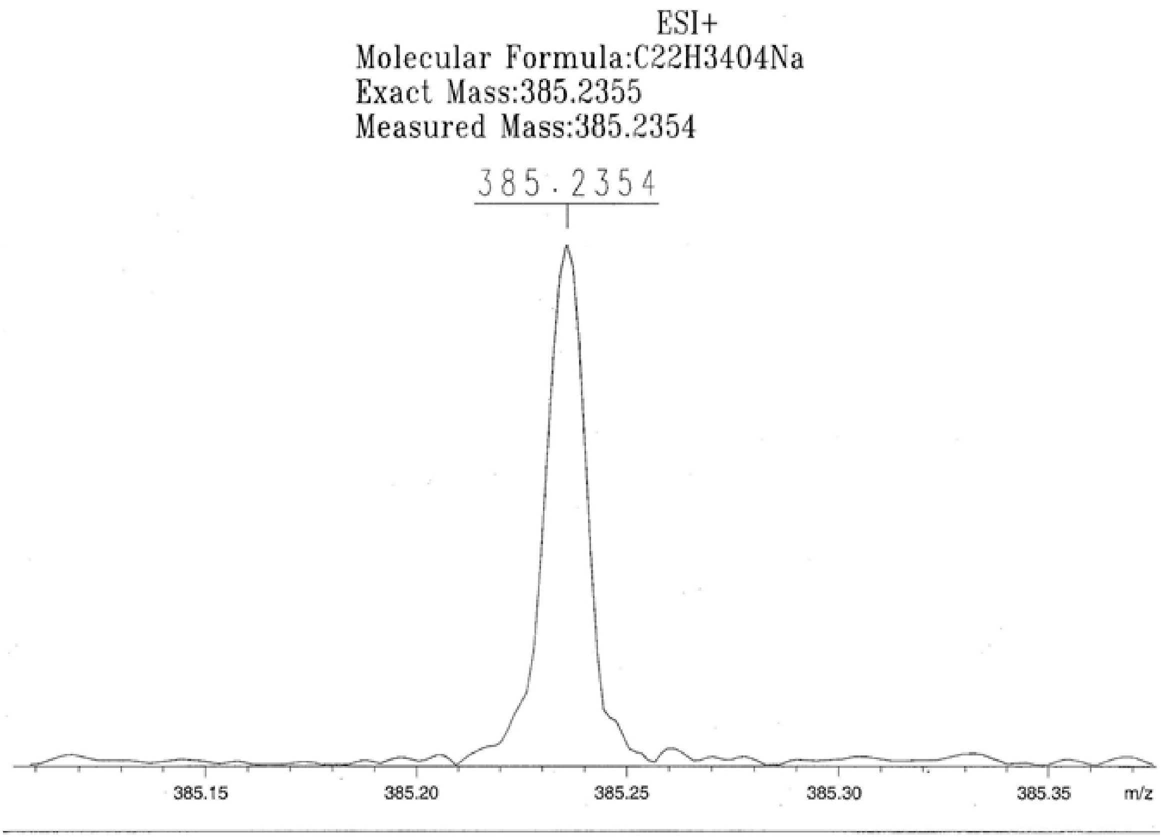

Figure S7. HRESIMS spectrum of **3**.

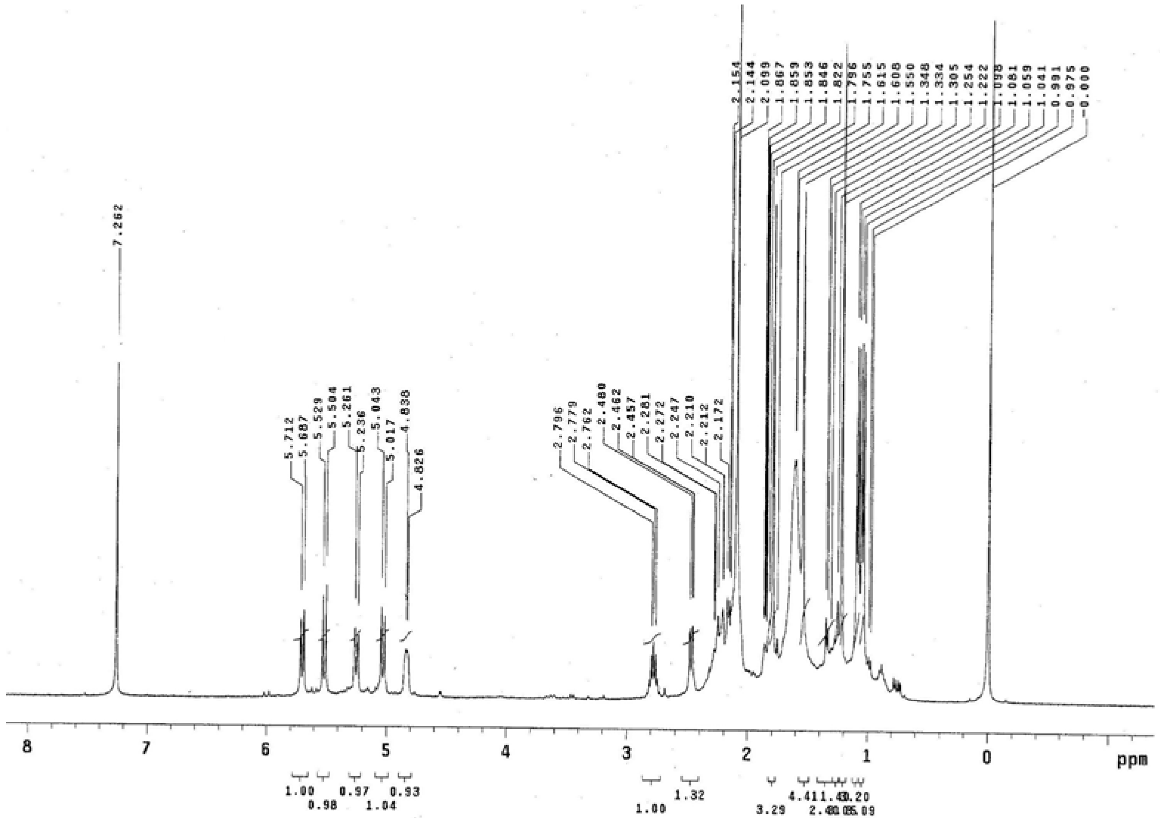

Figure S8. ^{1}H NMR spectrum of **3** in $CDCl_3$ at 500 MHz.

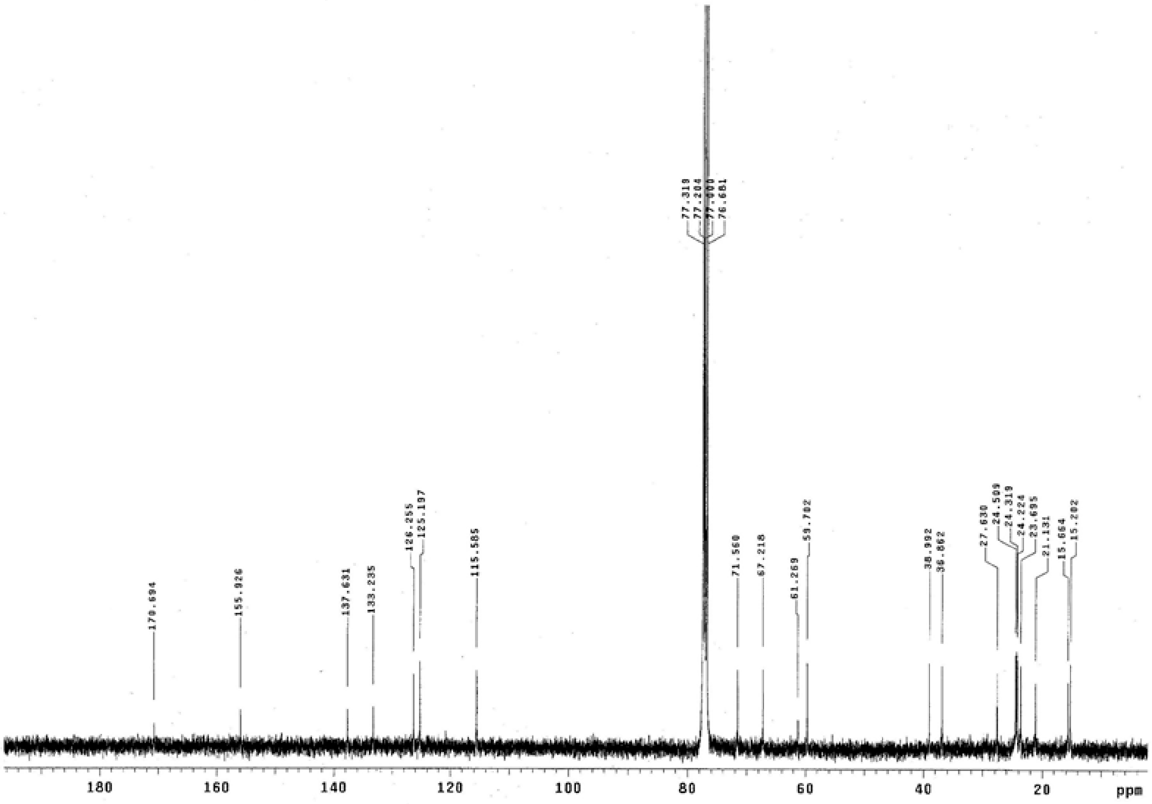

Figure S9. ^{13}C NMR spectrum of **3** in $CDCl_3$ at 125 MHz.

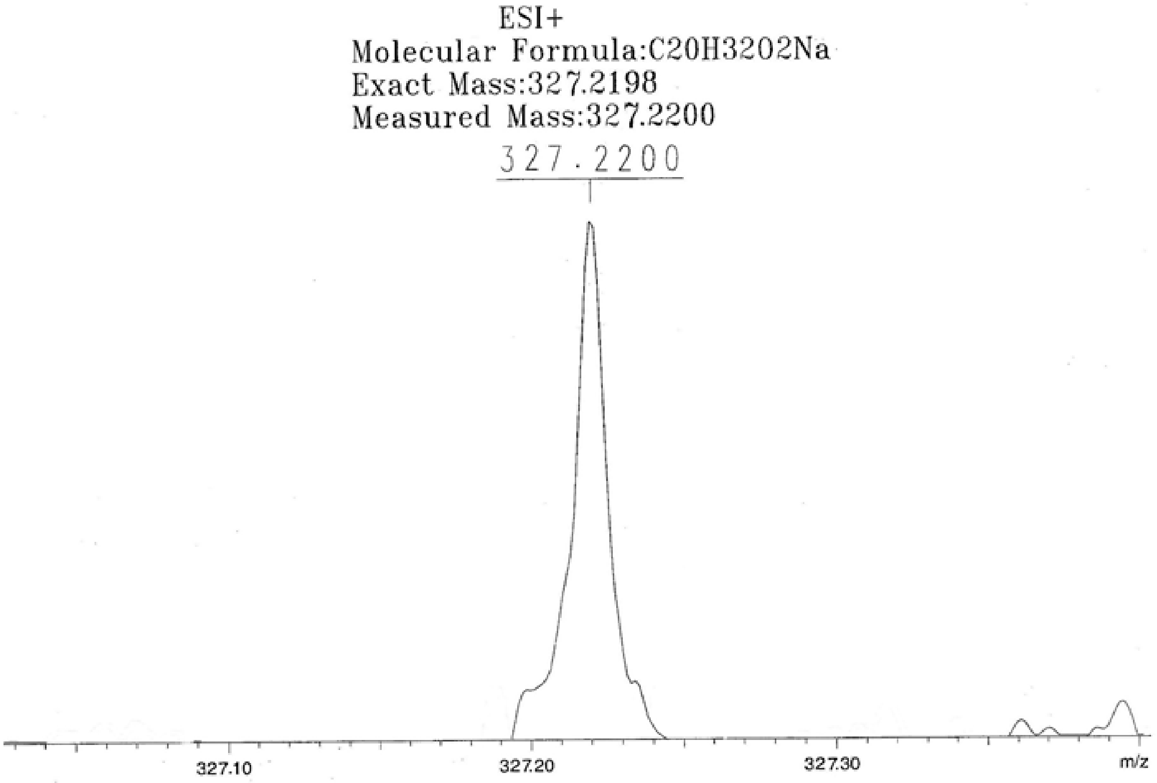

Figure S10. HRESIMS spectrum of **4**.

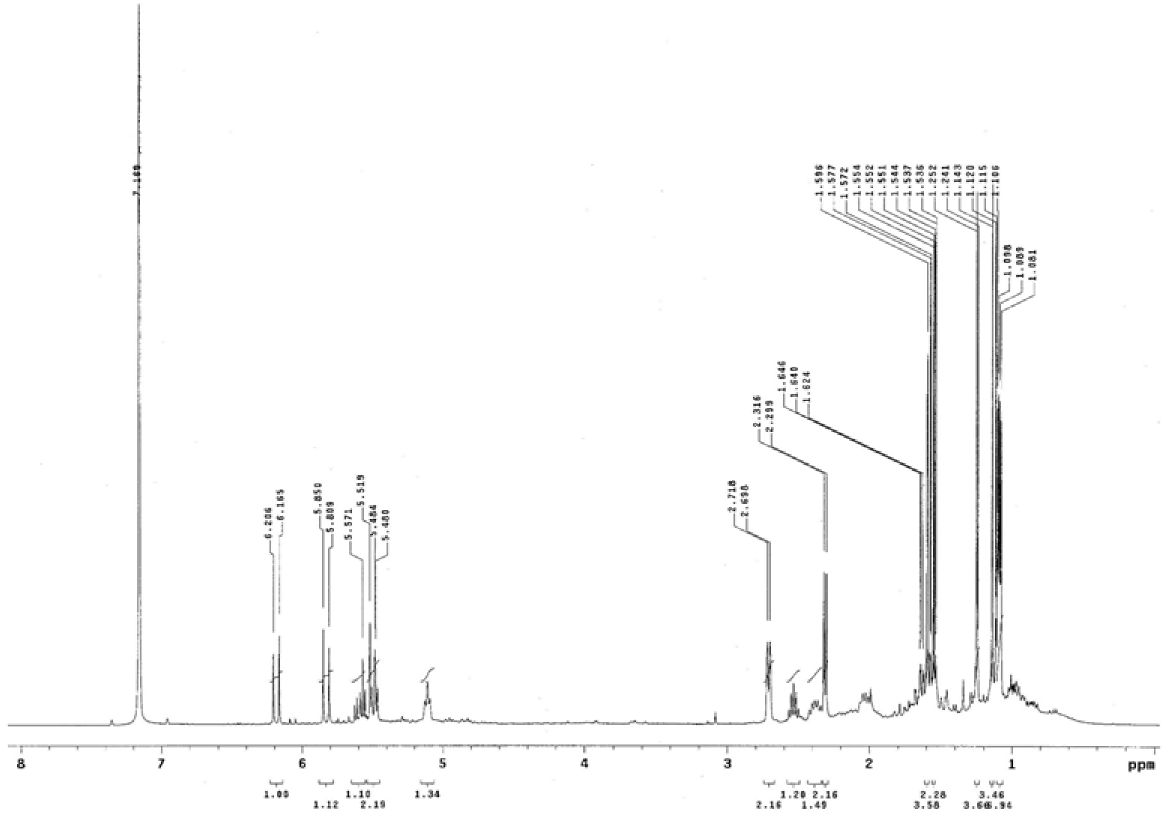

Figure S11. ^{1}H NMR spectrum of **4** in C_6D_6 at 400 MHz.

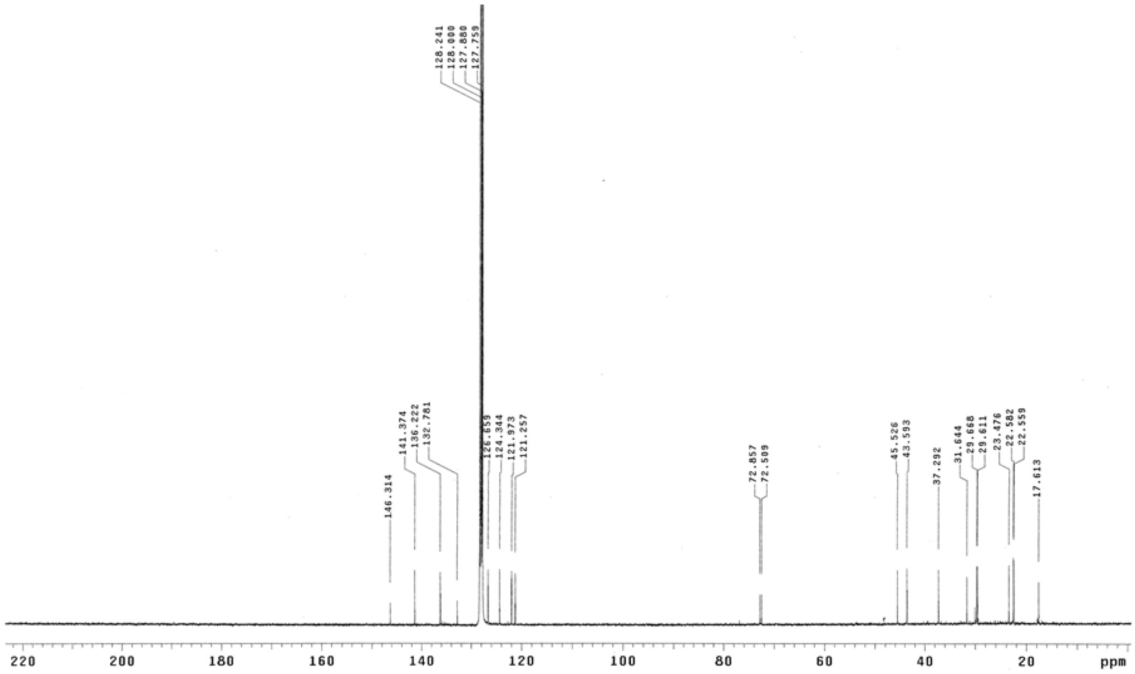

Figure S12. ^{13}C NMR spectrum of **4** in C_6D_6 at 100 MHz.

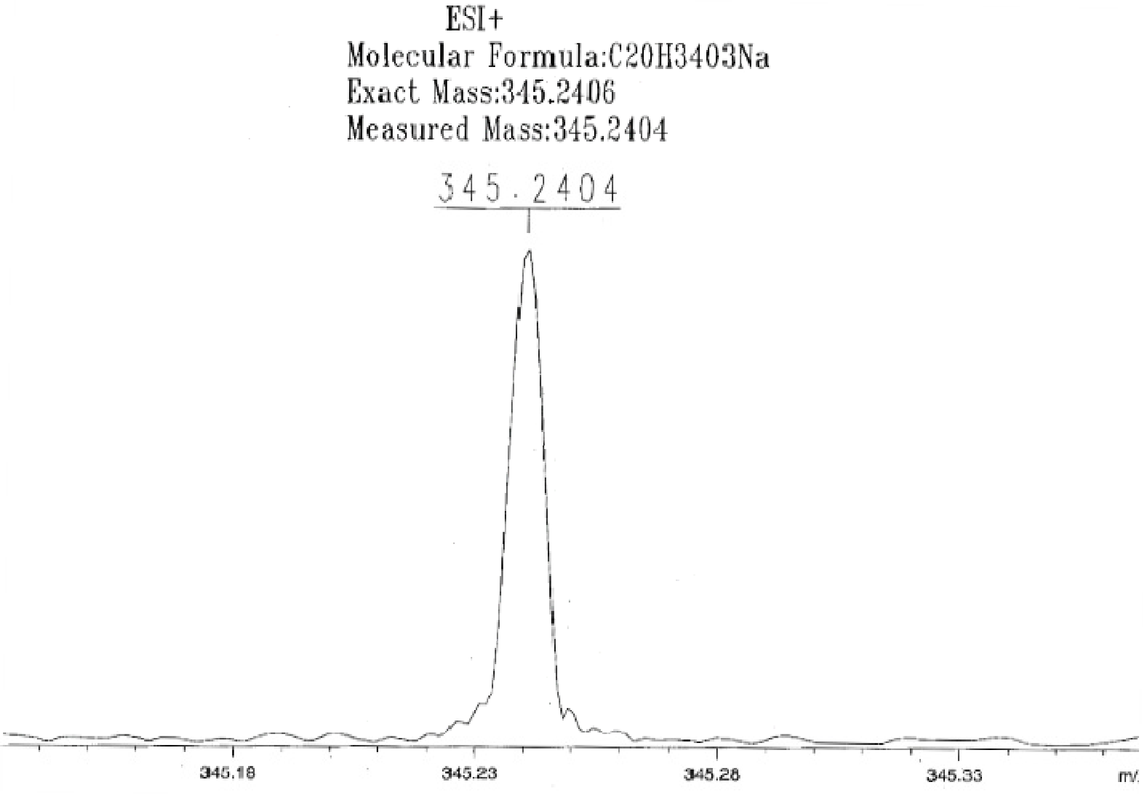

Figure S13. HRESIMS spectrum of **5**.

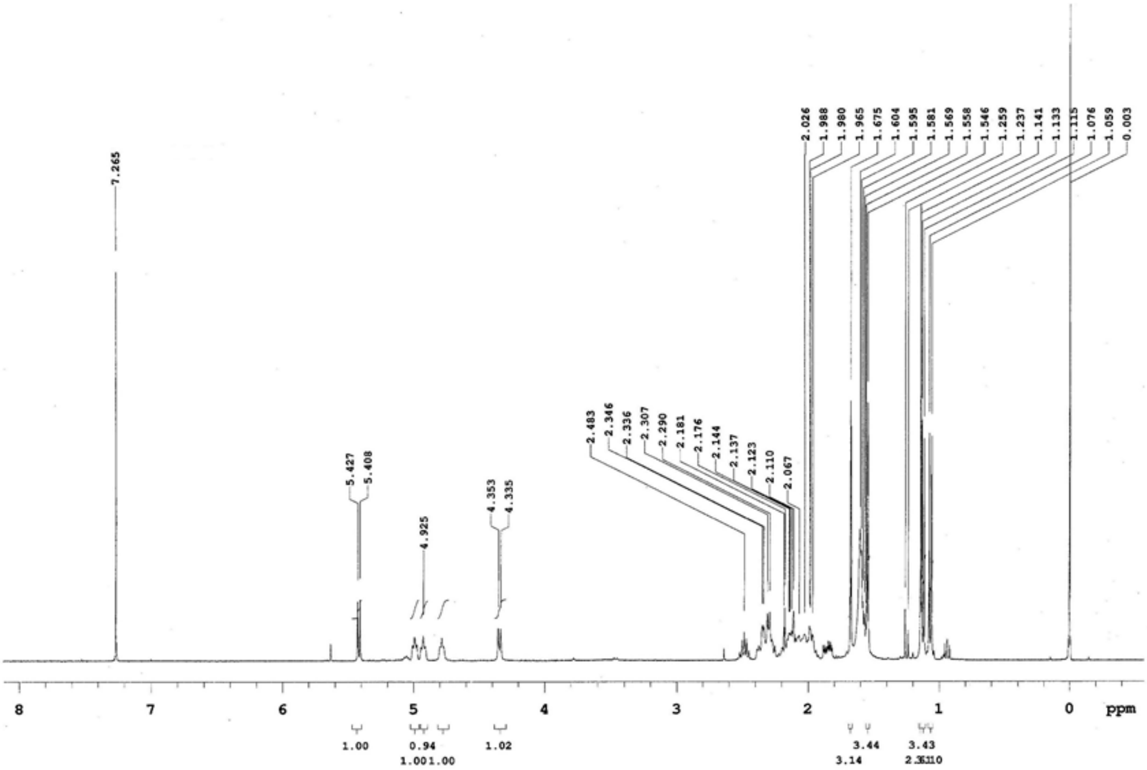

Figure S14. ^{1}H NMR spectrum of **5** in $CDCl_3$ at 400 MHz.

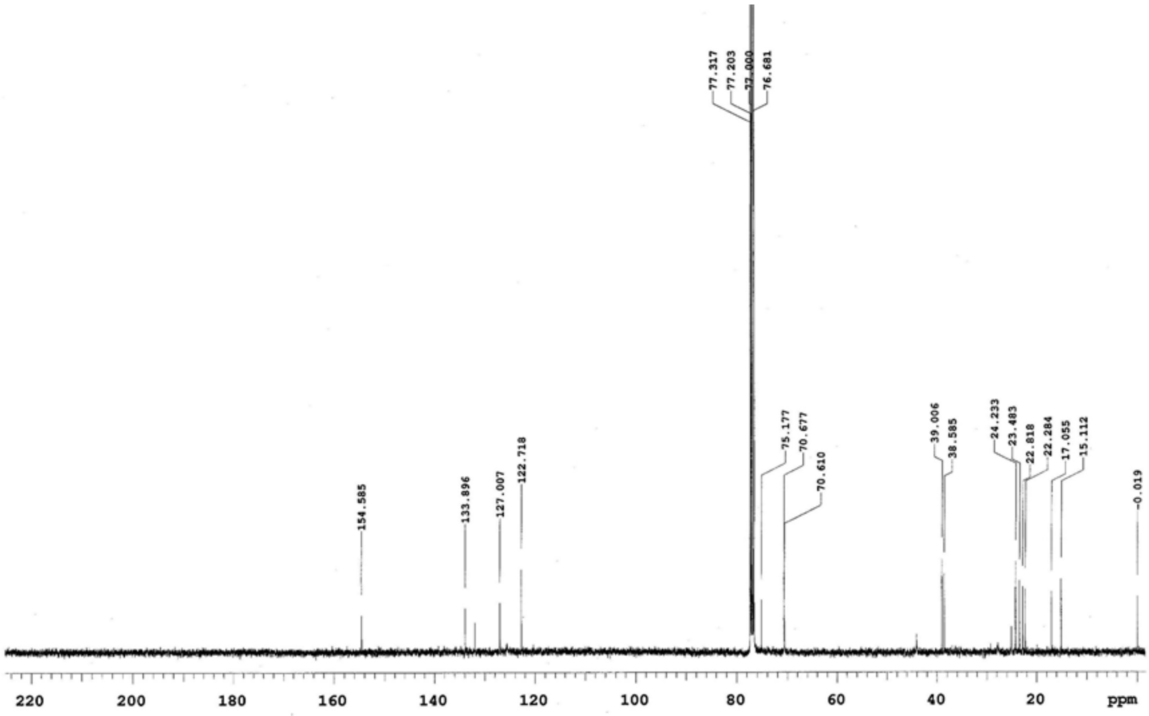

Figure S15. ^{13}C NMR spectrum of **5** in $CDCl_3$ at 100 MHz.

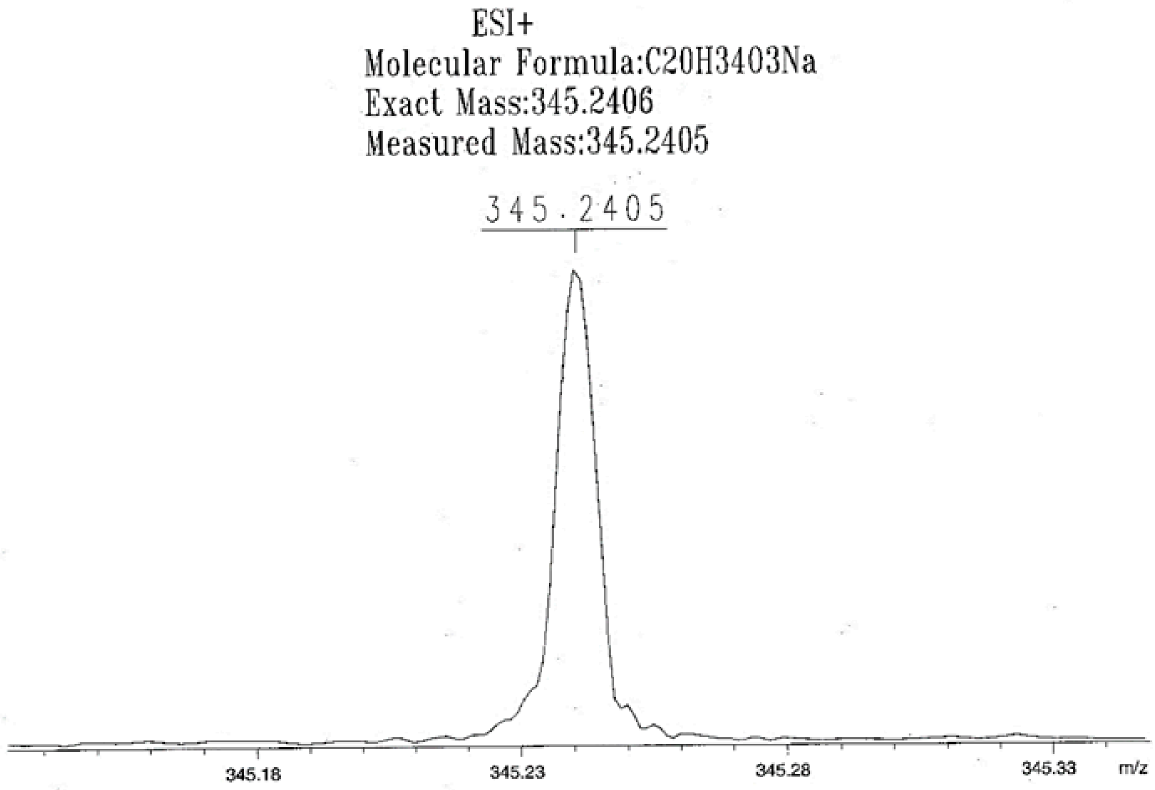

Figure S16. HRESMS spectrum of 6.

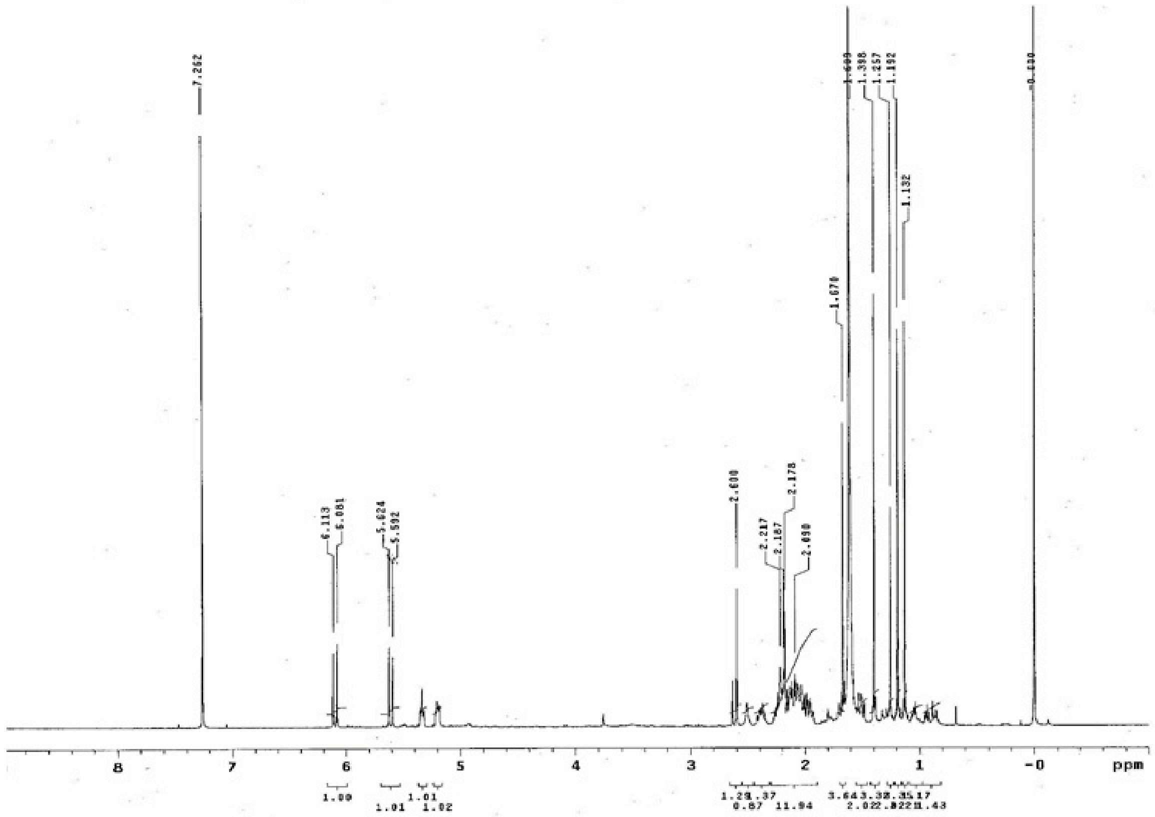

Figure S17. ¹H NMR spectrum of **6** in $CDCl_3$ at 500 MHz.

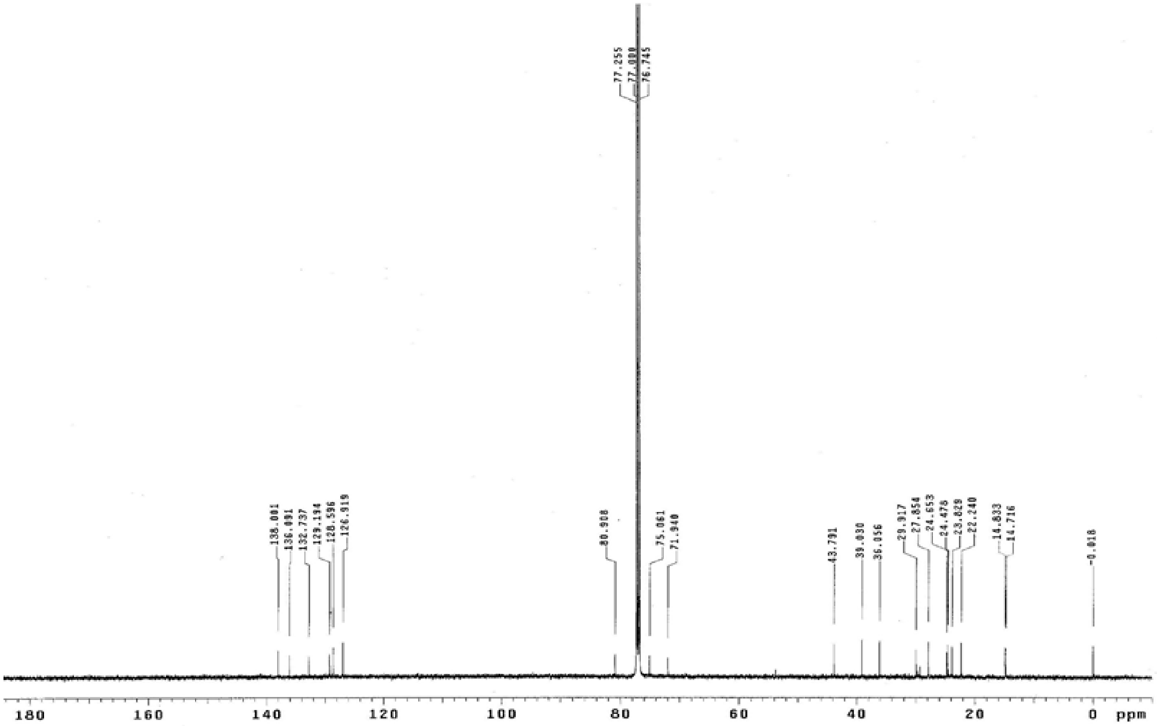

Figure S18. ^{13}C NMR spectrum of **6** in $CDCl_3$ at 125 MHz.

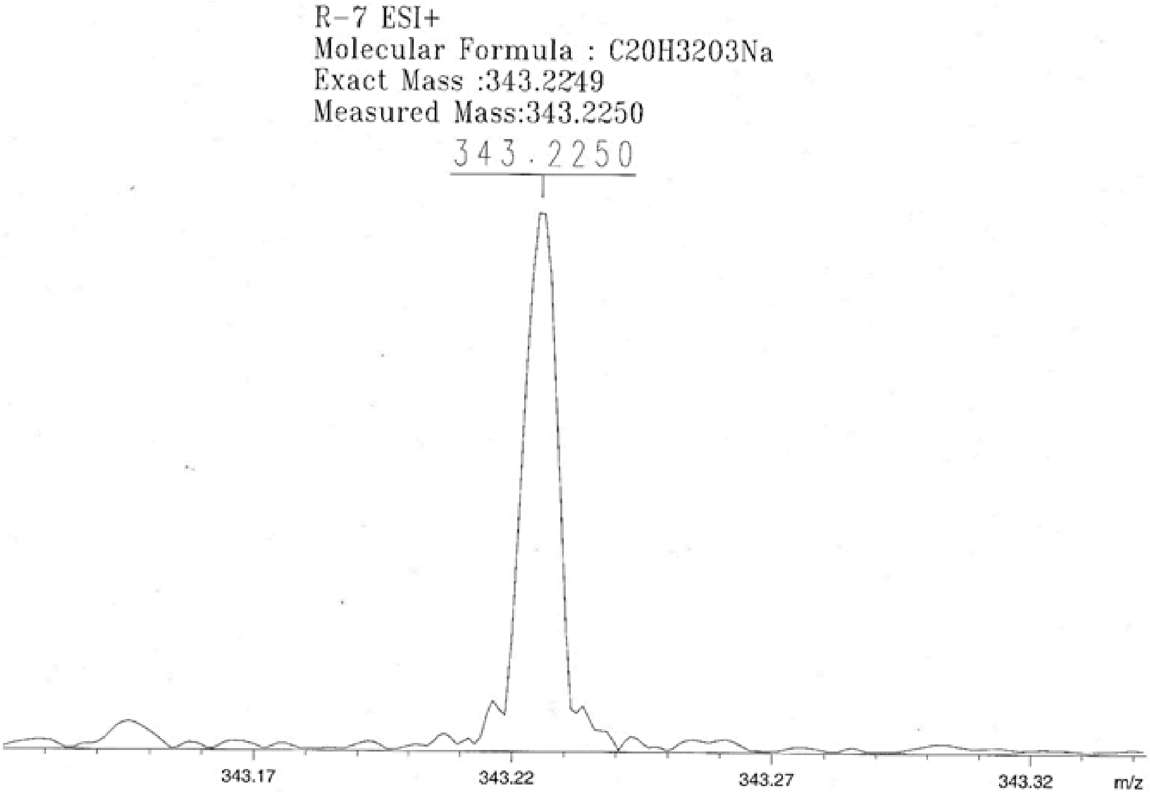

Figure S19. HRESIMS spectrum of **7**.

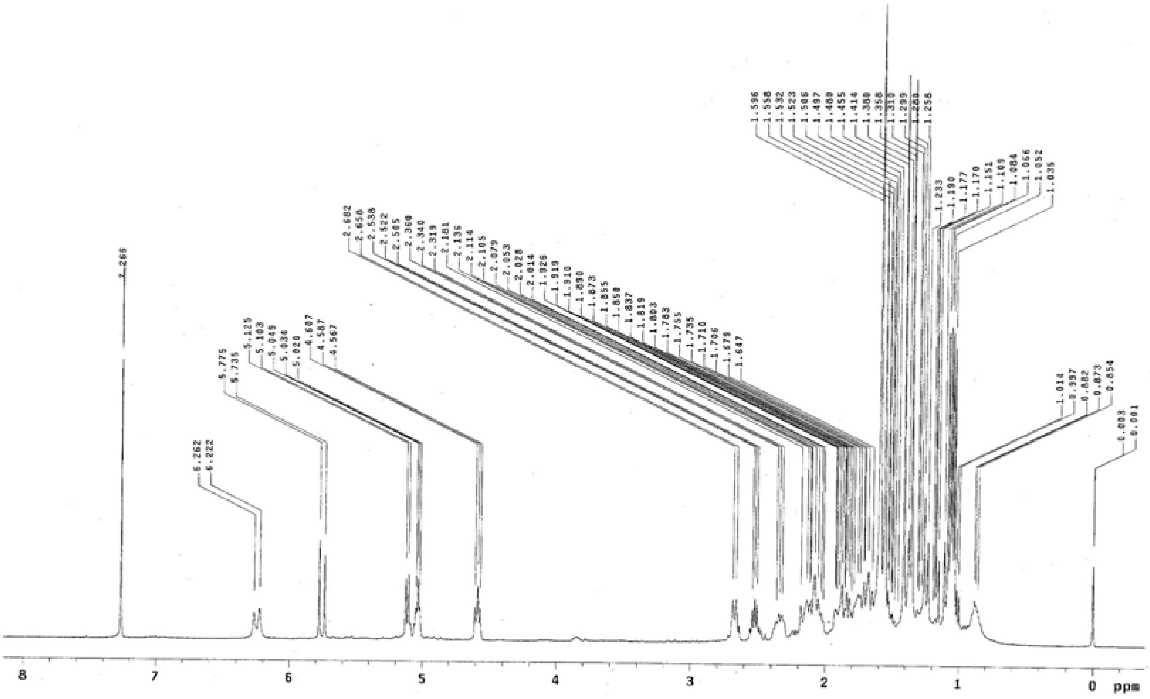

Figure S20. ^{1}H NMR spectrum of **7** in $CDCl_3$ at 400 MHz.

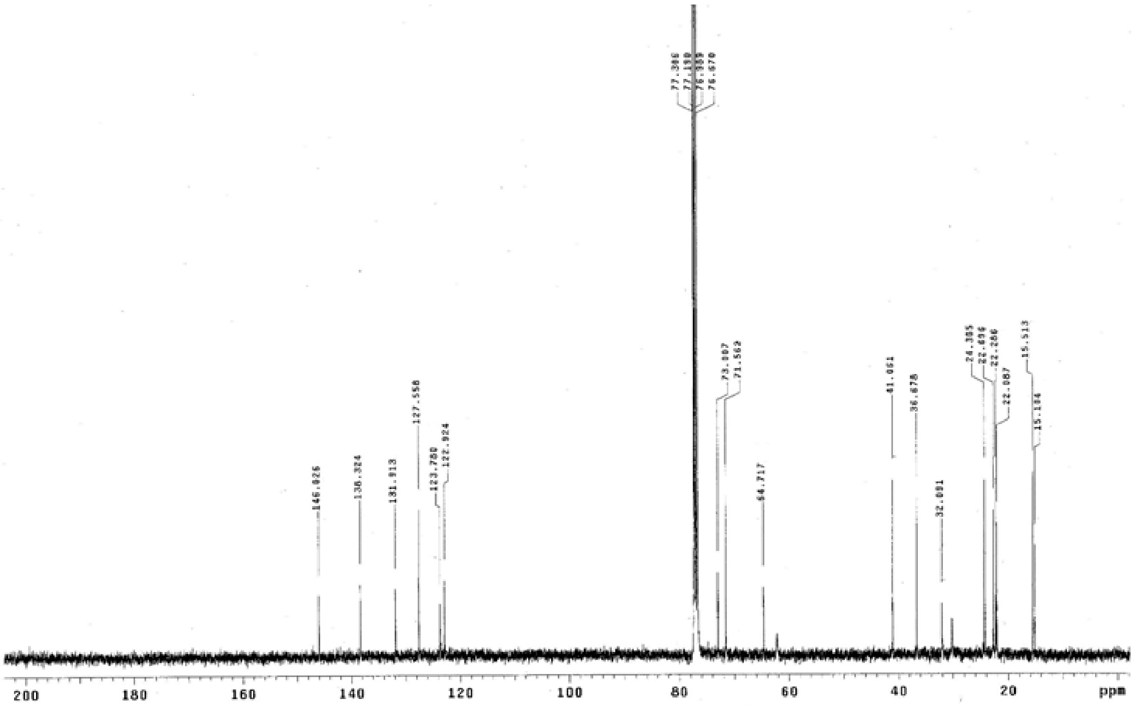

Figure S21. ^{13}C NMR spectrum of **7** in $CDCl_3$ at 100 MHz.

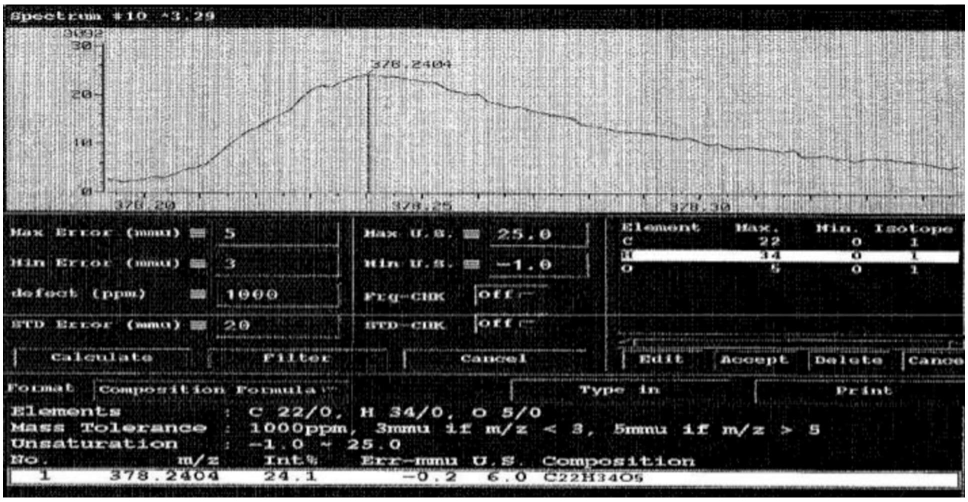

Figure S22. HRESIMS spectrum of **8**.

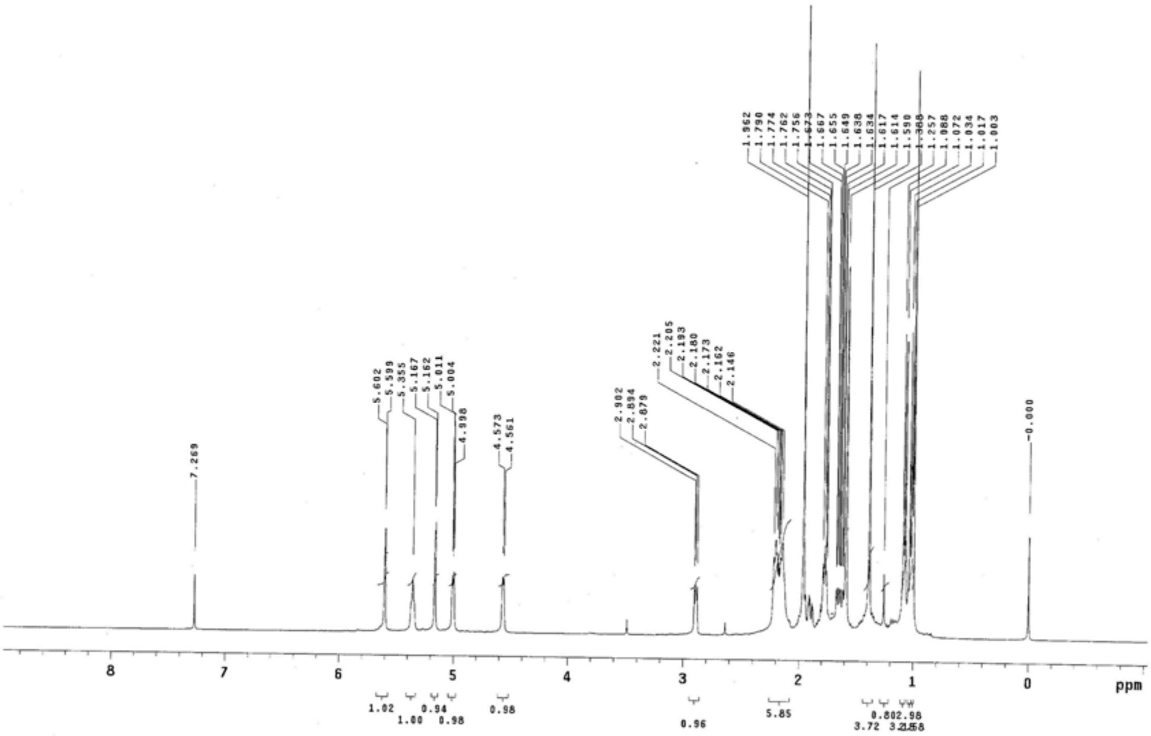

Figure S23. ^{1}H NMR spectrum of **8** in $CDCl_3$ at 400 MHz.

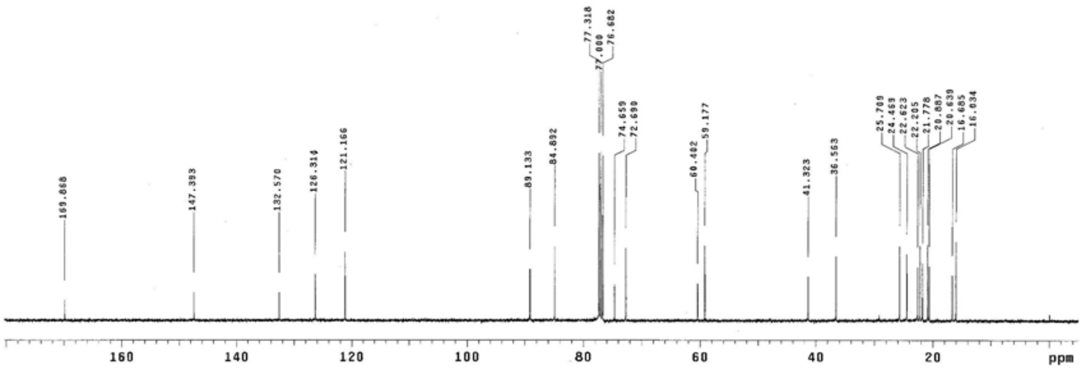

Figure S24. ^{13}C NMR spectrum of **8** in $CDCl_3$ at 100 MHz.

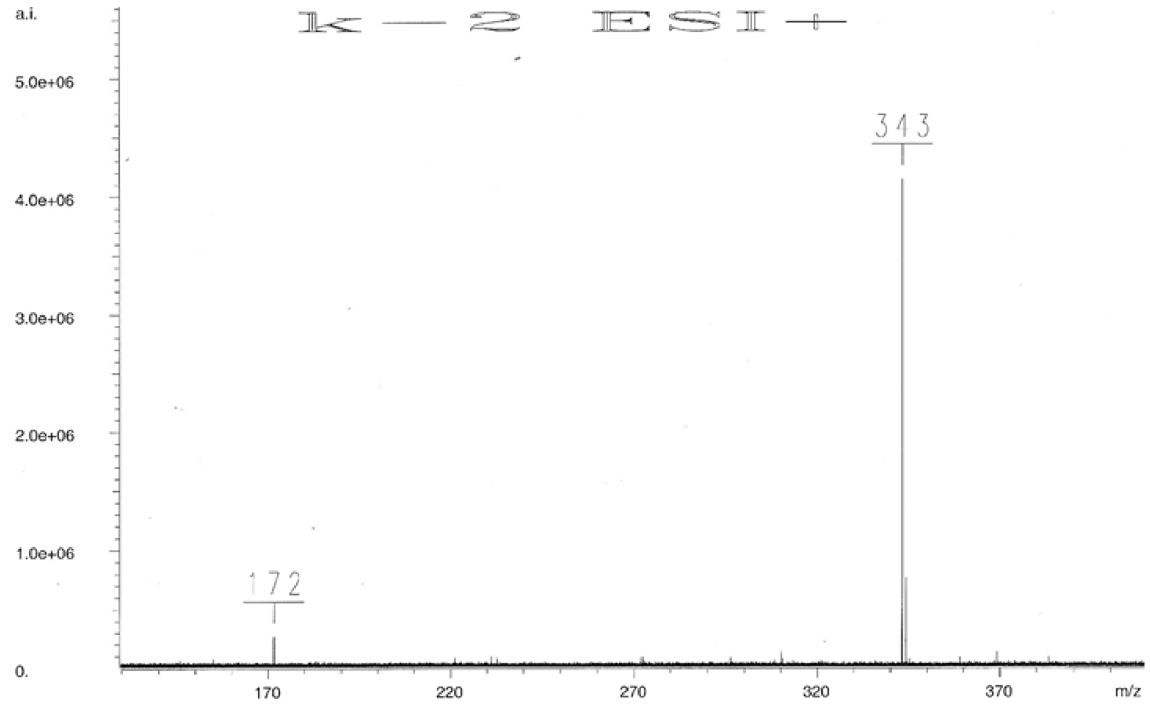

Figure S25. ESIMS spectrum of **9**.

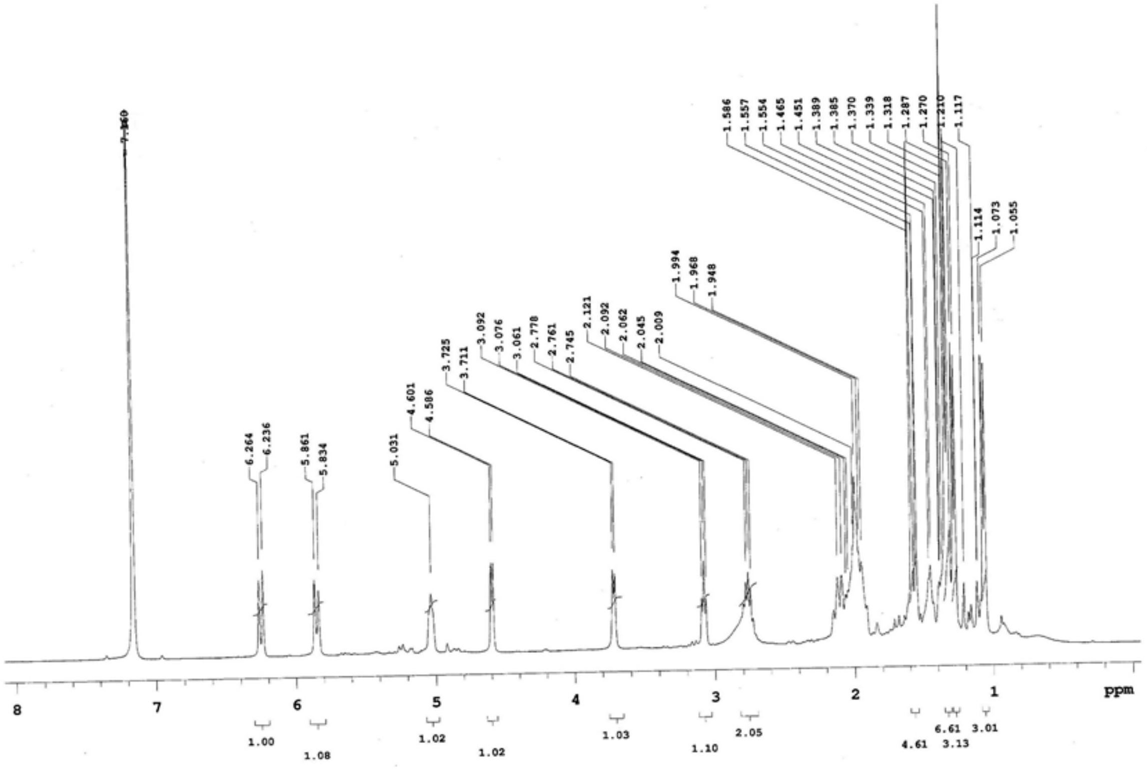

Figure S26. ^{1}H NMR spectrum of **9** in C_6D_6 at 400 MHz.

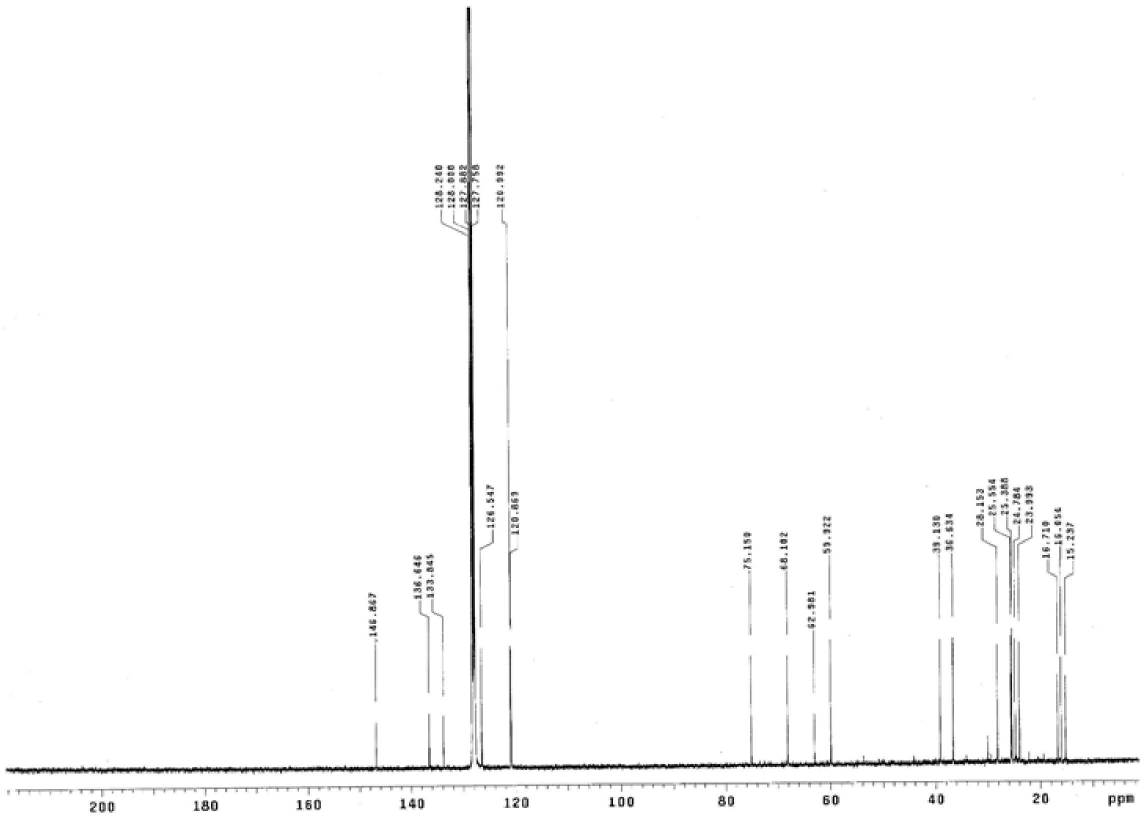

Figure S27. ^{13}C NMR spectrum of **9** in C_6D_6 at 100 MHz.
